# State-of-the-art polyetheretherketone three-dimensional printing and multifunctional modification for dental implants

**DOI:** 10.3389/fbioe.2023.1271629

**Published:** 2023-10-19

**Authors:** Meiqing Chen, Mei Ren, Yingqi Shi, Xiuyu Liu, Hongtao Wei

**Affiliations:** ^1^ Department of Stomatology, China-Japan Union Hospital of Jilin University, Changchun, China; ^2^ Hospital of Stomatogy, Jilin University, Changchun, China

**Keywords:** polyetheretherketone, dental implants, three-dimensional printing, functional modification, biological activity

## Abstract

Polyetheretherketone (PEEK) is a high-performance thermoplastic polymer with an elastic modulus close to that of the jawbone. PEEK has the potential to become a new dental implant material for special patients due to its radiolucency, chemical stability, color similarity to teeth, and low allergy rate. However, the aromatic main chain and lack of surface charge and chemical functional groups make PEEK hydrophobic and biologically inert, which hinders subsequent protein adsorption and osteoblast adhesion and differentiation. This will be detrimental to the deposition and mineralization of apatite on the surface of PEEK and limit its clinical application. Researchers have explored different modification methods to effectively improve the biomechanical, antibacterial, immunomodulatory, angiogenic, antioxidative, osteogenic and anti-osteoclastogenic, and soft tissue adhesion properties. This review comprehensively summarizes the latest research progress in material property advantages, three-dimensional printing synthesis, and functional modification of PEEK in the fields of implant dentistry and provides solutions for existing difficulties. We confirm the broad prospects of PEEK as a dental implant material to promote the clinical conversion of PEEK-based dental implants.

## 1 Introduction

In the field of dentistry, the primary goal is to reconstruct missing teeth to restore original function and maximize esthetic performance. With the rapid development of social economy and the improvement in esthetic concepts, dental implants—the rebirth of teeth—have become the most eye-catching repair option in stomatology. The superiority of dental implants has led to a mushroomed increase in their demand worldwide. It has been reported that the number of dental implants being used exceeded a million per year over a decade ago, and the global dental implant market is expected to reach $6.81 billion by 2024 (Jiang et al., 2020). The enormous demand of society and the limitations of existing materials have greatly promoted the interest in developing alternative materials for dental implants. Polyetheretherketone (PEEK) is a widely used standard biopolymer material for implantation. It has excellent esthetic properties, and the elastic modulus of reinforced PEEK can be comparable to that of human bones. A British scientist first produced PEEK in 1978, and in the 1980s, PEEK was approved by the US Food and Drug Administration (FDA) as an *in vivo* implantable material. It was also during this period that the role of material surface properties in osseointegration was revealed and recognized. Until April 1998, Thornton Cleveleys first proposed the use of implantable PEEK as a commercial biomaterial. Since then, the application of PEEK in biomedicine has grown exponentially, becoming a substitute for metal implants and the preferred material in plastic and trauma surgery (Mishra and Chowdhary, 2019; AlOtaibi et al., 2020; He et al., 2021; Ma et al., 2023). At present, PEEK is developed to be used not only as dental implants but also in the fields of maxillofacial/cranial implants, general orthopedic surgery, spinal surgery, and cardiac surgery (Panayotov et al., 2016).

The key factor for successful dental implantation is excellent osseointegration with surrounding bone tissues. Osseointegration, proposed by R. Branemark, refers to the direct contact and mechanical bonding between dental implants and the jawbone without any interference from fibers or connective tissue (Kadambi et al., 2021). Clinically, osseointegration is defined as the asymptomatic rigid fixation of allograft implants in bone under functional load. In 2017, a study described osseointegration as a response to isolating foreign implants (AlOtaibi et al., 2020). By dividing molecular and cellular communication of post-implantation, osseointegration is divided into four stages: homeostasis, the inflammatory phase, the proliferative phase, and the remodeling phase (Pidhatika et al., 2022). Effective osseointegration provides support and structural stability for dental implants. However, as PEEK does not have bone induction ability in essence, it lacks the capacity to induce osteogenic differentiation and stimulate new bone formation, which affects osseointegration between PEEK implants and the surrounding alveolar bone. Therefore, PEEK urgently requires modification methods to improve its biological activity to achieve osseointegration.

**FIGURE 1:**
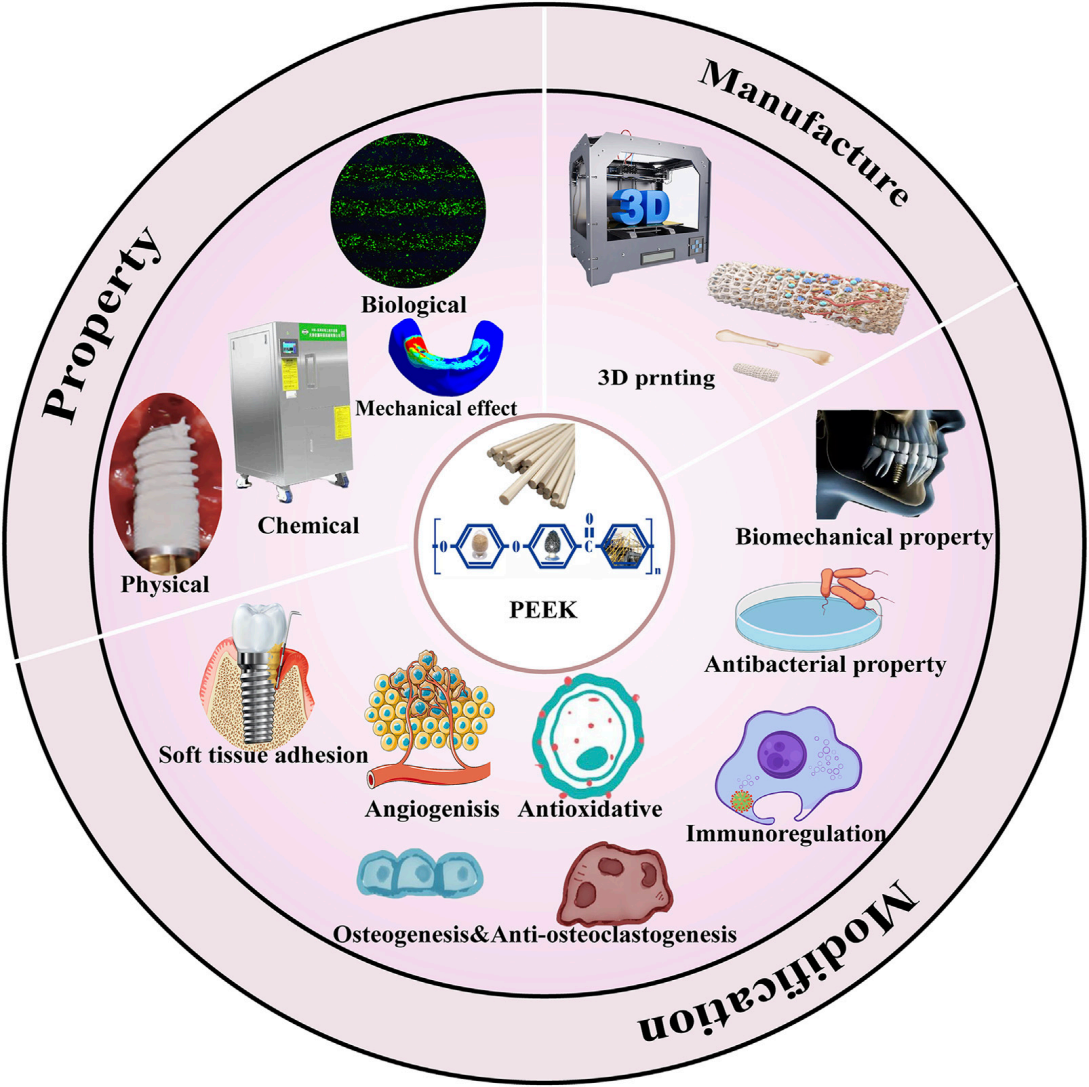
PEEK property, manufacture, and modification.

This paper provides a detailed comparison of traditional metal implant materials and PEEK in terms of physical, chemical, and biological properties for the first time. Three-dimensional finite element analysis (FEA) also indicates the mechanical advantages of PEEK at the implant–bone interface. The aforementioned points confirm the feasibility and reasons of PEEK being used as a novel implant substitute material. Second, we introduce the most suitable method for manufacturing PEEK dental implants—3D printing—and discuss methods to solve existing research hotspots in 3D printing, such as the research progress in improving the interlayer adhesion of 3D-printed PEEK. In addition, as an important aspect, we comprehensively summarize different modification strategies for improving the biomechanical properties, antibacterial properties, soft tissue adhesion ability, immunoregulation ability, and antioxidative, osteogenic and anti-osteoclastogenic, and angiogenic properties of PEEK. This review aims to comprehensively analyze in detail the clinical application potential of PEEK-based dental implant materials and summarize the latest progress in improving their different activities from a functional perspective. Figure 1 summarizes the article content.

## 2 Property advantages of PEEK

### 2.1 Limitations of metal implants

Currently, the vast majority of dental implants are made of metals, such as titanium (Ti) and Ti alloys (principally Ti6Al4V)—the current gold standard in dentistry. However, the high elastic modulus of Ti affects the stress adjustment of bone tissue and a series of problems, which eventually cause stress shielding (Sumner, 2015). Since the elastic modulus of a Ti implant is more than five times that of cortical bone (14 GPa), most of the pressure will be transferred to the implant and reduce the load of cortical bone; according to Wolff’s law, there is a positive correlation between the load and bone growth, so it will gradually lead to the decrease in bone mineral density and chronic osteoporosis (Liu et al., 2021a; Kruse et al., 2021; Manzoor et al., 2021). Marginal bone loss of Ti implants can reach a certain extent after the first year of stress (Najeeb et al., 2016b). Therefore, the mechanical binding of the bone–implant interface is affected, and the implant becomes loose, leading to implantation failure (Nagels et al., 2003). Some other limitations of Ti have been found in clinical use and manufacturing, as given in Table 1.

**TABLE 1:** Limitations of Ti.

Limitations	Consequence/mechanism	References
Common metal problems—wear, metal fatigue, and fretting corrosion	Affect its service life	Guo et al. (2022b)
Metallic heterocurrent	When other kinds of metals are around in oral, it is formed	da Cruz et al. (2021)
Metal allergy (rate is about 0.6%)	It can cause cellular sensitization; clinical manifestations are hypersensitivity and anaphylaxis; specific manifestations can be eczema, urticaria, edema, pruritus of skin, facial erythema, necrosis, pain, and hyperplastic tissues	Sicilia et al. (2008); Guo et al. (2021a)
Galvanic corrode	It slowly leaks out trace amounts of elements that can be toxic to humans, such as aluminum and vanadium. At the same time, the release of Ti ions can upregulate the pro-inflammatory factor, inhibit the activity of osteoblasts, promote osteoclast activity, and change the microbial composition of the biofilm then cause peri-implantitis	Ren et al. (2020a); Kim et al. (2019); Noronha Oliveira et al. (2018); Alves et al. (2022)
Metallic color and gray appearance	It can be seen in thin gingival types or high-smile line patients, which greatly affects the esthetic effect that has been valued at present	Azizi et al. (2018); Schwitalla and Müller (2013)
Metal design limitations	Slender central screw used to connect the abutment to the metal implant is prone to fatigue and fracture when subjected to non-axial forces and cyclic occlusion. At this time, the rigid Ti implant cannot drill a hole with a dental drill to remove the snapped screw	Jiang et al. (2020)
Density of 4.506 g/cm^3^	It is about three times the average human bone density	Fu et al. (2021a)
Poor biological activity, slow healing, porosity, thermal and electrical conductivity, complex manufacturing processes, and expensive production costs	-	Sagomonyants et al. (2008); Carpenter et al. (2018); Jovanović et al. (2021)

### 2.2 Property advantages of PEEK as an implant

**FIGURE 2:**
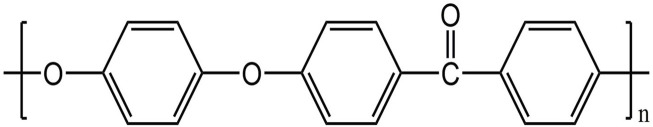
Molecular structure of PEEK (Ma et al., 2020).

Because of the defects of metal and ceramic-based materials, there is an urgent need for a new implant alternative multifunctional material to adapt to the rapid development of material and oral medical systems. Poly(oxy-1,4-phenyleneoxy-1,4-phenylenecarbonyl-1,4-phenylene), abbreviated as PEEK, combines excellent physical, chemical, and biological properties (Searle and Pfeiffer, 1985). PEEK is a leading high-performance thermoplastic polymer in the polyaryl-ether-ketone (PAEK) family used in dental biomedical sciences (Jung et al., 2019; Araújo Nobre et al., 2021). Compared with traditional thermosets such as epoxy, thermoplastics not only have equivalent mechanical properties but also have low manufacturing time and cost, high toughness, less crosslinks, easier repair and recycling, and easily undergo secondary processing through melting and reconsolidation (Boon et al., 2021; Chen et al., 2021). The sequence and proportion of phenylene rings (aryl), carbonyl sets (R-CO-R), and ether bonds (R-O-R) in the molecular backbone of the PAEK family are different. Flexibility and rigidity are provided by R-O-R and R-CO-R, respectively, which enhance the inter-molecular interactions and aryl (Toth et al., 2006; Alqurashi et al., 2021). The main chain of PEEK is composed of repeating units of a single ketone bond and double ether bonds (Figure 2). As we all know, structure determines property. The structure of PEEK not only makes it the only polymer material that can support repetitive loading without plastic deformation and fracture (Yakufu et al., 2020) but also makes it highly hydrophobic (Lee et al., 2012).

As a special organic plastic, it is tooth-colored, which meets esthetic requirements, and overcomes the problem of gingival staining caused by alloys (Najeeb et al., 2016b; Benli et al., 2020). When the implant is screwed into bone tissue or subjected to complex occlusion force, it experiences wear and tear, and excellent abrasion resistance can reduce the production of fragments that stimulate surrounding tissues and cause inflammation. Chen et al. (2020b) prepared PEEK coating on a central screw thread through thermal spraying and measured the friction coefficient and clamping force of the screw thread pair. It turned out that PEEK coating could reduce the friction coefficient and increase clamping force and preload under large-scale sliding, improving anti-loosening performance of screws under dynamic load. The aforementioned results show that PEEK has good abrasion resistance. PEEK is radiolucent and does not affect the nuclear magnetic resonance imaging (MRI) examination, so the healing of soft and hard tissues around the implant can be clearly observed (Korn et al., 2015). Köse et al. (2021) produced a polymethylmethacrylate phantom and placed prosthetic material cylinders (Co-Cr, Ti, zirconia, and PEEK) into a hole to compare artifacts in cone beam computed tomography (CBCT) images. They evaluated the presence of artifacts by calculating the standard deviation (SD) of grayscale values in regions of interest (ROIs) around each material. Since PEEK has the lowest density and atomic number, it absorbs the least radiation. The experimental result proved that the artifacts of PEEK are similar to the empty phantom (control group), which are significantly lower than those of the zirconia, Co-Cr, and Ti groups (*p* < .05). From the perspective of precise radiographic images, PEEK is the preferred dental implant material. Natural periodontal ligament has shock absorption function, and there is a lack of similar tissue around the implant and direct contact with bone tissue. After absorbing the occlusion force received, PEEK gently and smoothly transmits it to the surrounding bone tissue, thereby achieving shock absorption and protecting bone tissue from heavy loads to avoid implant failure, which can extend the lifespan of the implant (Anguiano-Sanchez et al., 2016; Zhao et al., 2020). Significantly, the long-term success rate of implants depends on whether they can minimize marginal bone loss after functional loading. According to Hooke’s law, similar mechanical properties of PEEK and bone distribute stress evenly and they share a similar amount of modulus to minimize disuse bone resorption caused by stress shielding, which also improves implant stability by enhancing the bond between implants and bone (Steenberghe et al., 2001; Chen et al., 2012; Jiang et al., 2020). Interestingly, a similar elastic modulus can also be used to provide a damping effect for PEEK restorations (Papathanasiou et al., 2020). Research has shown that carbon fiber-reinforced PEEK (CFR-PEEK) can withstand maximum chewing pressure (306 N) under oral physiological conditions (Wang et al., 2023). The specific strength (strength-to-weight ratio) of pure PEEK also gives it excellent mechanical strength (Marin et al., 2020). PEEK also has excellent chemical stability, thermal stability, and biocompatibility, which can be proved by resisting all chemical reagents except 98% sulfuric acid, and long-term stable mechanical performance in a 120 °C environment (Kurtz and Devine, 2007). In a previous study, PEEK resisted *in vivo* degradation and damage simulated by lipid exposure (AlOtaibi et al., 2020). In addition, during the initial healing phase, a clinical controlled trial using Ti and polymer abutments did not show an increasing risk of marginal bone resorption or soft tissue decline (Koutouzis et al., 2011). These properties are shown in Table 2. In addition, creep resistance, nonmagnetic property, high bending and compression resistance, no exothermic reaction, low solubility and water absorption, and self-lubrication also make it an attractive biological engineering material (Wang et al., 2023).

**TABLE 2:** Chemical and biological properties of PEEK.

	Property advantages	Application significance	References
Chemical properties	Resistance to chemical, thermal, and bio-degradation	It can be applied to complex oral environments through various manufacturing methods	Fu et al. (2021a)
	Superior processability	It enables PEEK to accurately manufacture various complex structures of implants	Ma et al. (2020)
	Resistance to ethylene oxide gas, γ radiation, and steam	It can withstand repeated sterilization	Singh et al. (2019)
Biological properties	PEEK has a two-phase semi-crystalline structure which does not provide any kind of cytotoxicity or mutagenicity	It shows high compatibility with soft and hard tissues	Lethaus et al. (2012); Maloo et al. (2022)
	Low plaque affinity	Inhibition of peri-implant inflammation	Najeeb et al. (2016b)

Due to the continuous and irreversible impact of stress and strain on the microstructure of alveolar bone, osseointegration is strongly influenced by the stress and strain distribution of the implant–bone interface, which is a key factor for long-term success in implantation (Bins-Ely et al., 2020). FEA is a method used to predict stress and strain at any point in any given geometric shape via theoretical models (Chokaree et al., 2022). In the field of stomatology, FEA has been recognized as a well-established research method for predicting the von Mises equivalent stress and strain, compressive/tensile stress, and strain energy density (SED) of various dental implants and peri-implant alveolar bone (Frost, 2004). Implant prosthesis repair of mandibular edentulous patients is usually a mixed-support All-on-4 treatment, consisting of a bar, top cap and four implants, screws, and abutments (Maló et al., 2003). Shash et al. (2022) found that the stress and strain changes in PEEK implants and surrounding bone tissue were smaller than those in Ti, so the chewing force on mixed-support dentures could be transferred to acrylic dentures and mucosa. The maximum von Mises stress (σM) also did not exceed the pain threshold (0.63–1.2 MPa) and yield strength (140–170 MPa), which would not cause part breakage and mucosal pain. Therefore, PEEK is more suitable for use as an All-on-4 implant, which can reduce the burden on the implant and alveolar bone, especially in the cases of poor bone quality. For the impact of the implant itself, the von Mises in CFR-PEEK implants will not exceed its ultimate strength, so there is no risk of fracture or yielding (Al-Mortadi et al., 2022). In addition, von Mises stresses of Ti implants are prone to concentrate in the neck, which may lead to postoperative fracture, while CFR-PEEK with Young’s modulus 19 GPa can avoid such problems (Sarot et al., 2010). For the implant–bone interface, Lee et al. (2012) found that 5*0.5 mm^2^ PEEK coating could increase the SED level of bone tissue around the implant, thus having a smaller stress-shielding effect compared to Ti and zirconium.

In summary, PEEK is a new implant material that can potentially replace Ti and zirconia, with reasons including improving aesthetics, reducing risks caused by mechanical properties, higher design freedom, reducing system costs, and more optional manufacturing methods (Shash et al., 2022). Although Ti and zirconia are the best known implant materials in dental applications among the biomedical alloys, PEEK takes it to the next level in some special circumstances, such as bruxism, metal allergy, and higher esthetic requirement (Lijnev et al., 2022).

## 3 Manufacture of PEEK implants—3D printing

In order to further improve the bioactivity and performance of implants, scholars start from the manufacturing process to search for suitable methods and parameters. Aiming to improve the efficiency of manufacturing oral restoration and the size accuracy of implants, while reducing the workload of dentists, a fully digital product manufacturing process—additive manufacturing (AM) technology (also known as 3D printing technology)—was created (Barazanchi et al., 2017). Such a digital procedure uses data flow to integrate disease diagnosis, treatment planning, and prosthesis production (Salmi, 2021). The working principle of AM is discrete stacking. The continuous superposition of a discrete process is transformed into a 3D digital model of a two-dimensional sheet model, and the entire process is sequentially stacked layer by layer by a computer program (Kessler et al., 2020). AM is not a type but a class of technology, and seven 3D printing categories are included in the American Society for Testing and Materials classification standard. Traditional subtractive machining technology (also known as numerical control processing technology, NC) of PEEK includes injection molding, thermal compression molding, and computer-aided design and computer-aided manufacturing (CAD/CAM) milling. Injection molding injects PEEK and/or its composites into a pre-designed mold under highly evaluated temperature and shear force to form a product with customized geometry. As for thermal compression molding, heat and pressure are applied to the molten state of PEEK in a platen mold of a given thickness to form products (He et al., 2021). Compared with NC, AM is suitable for the mass production of complex implants in a short period of time and can also reuse unformed raw materials to reduce costs (Tian et al., 2021). Among AM, commonly used methods in the field of PEEK oral implants include selective laser sintering (SLS) and fused filament fabrication (FFF) or fused deposition modeling (FDM) (Huang et al., 2023). SLS has the longest using time and the greatest potential for large-scale production (Luo et al., 2023). It selectively melts PEEK powder at high temperatures (i.e., > Tm of PEEK) generated by laser or electron beam irradiation, and the solid structure is fused together in a layer-by-layer manner (Ligon et al., 2017). Nevertheless, SLS wastes a large amount of PEEK powder, which may be potentially contaminated and cannot be reused as raw material. It also requires additional safety measures (Rodzeń et al., 2021b). Due to minimal waste and easy operation, FDM is considered the best printing method for PEEK. In FDM, PEEK powders are spun into filaments as raw material by FFF, and then, the molten filaments are extruded through the orifice of the nozzle and merged with the previously deposited material to form predetermined 3D porous scaffolds (Yang et al., 2017). Unfortunately, the PEEK scaffold generated by FDM has weak interior bonding strength and mechanical properties. The solutions are introduced in the following.

### 3.1 Solutions to enhance mechanical properties

**FIGURE 3:**
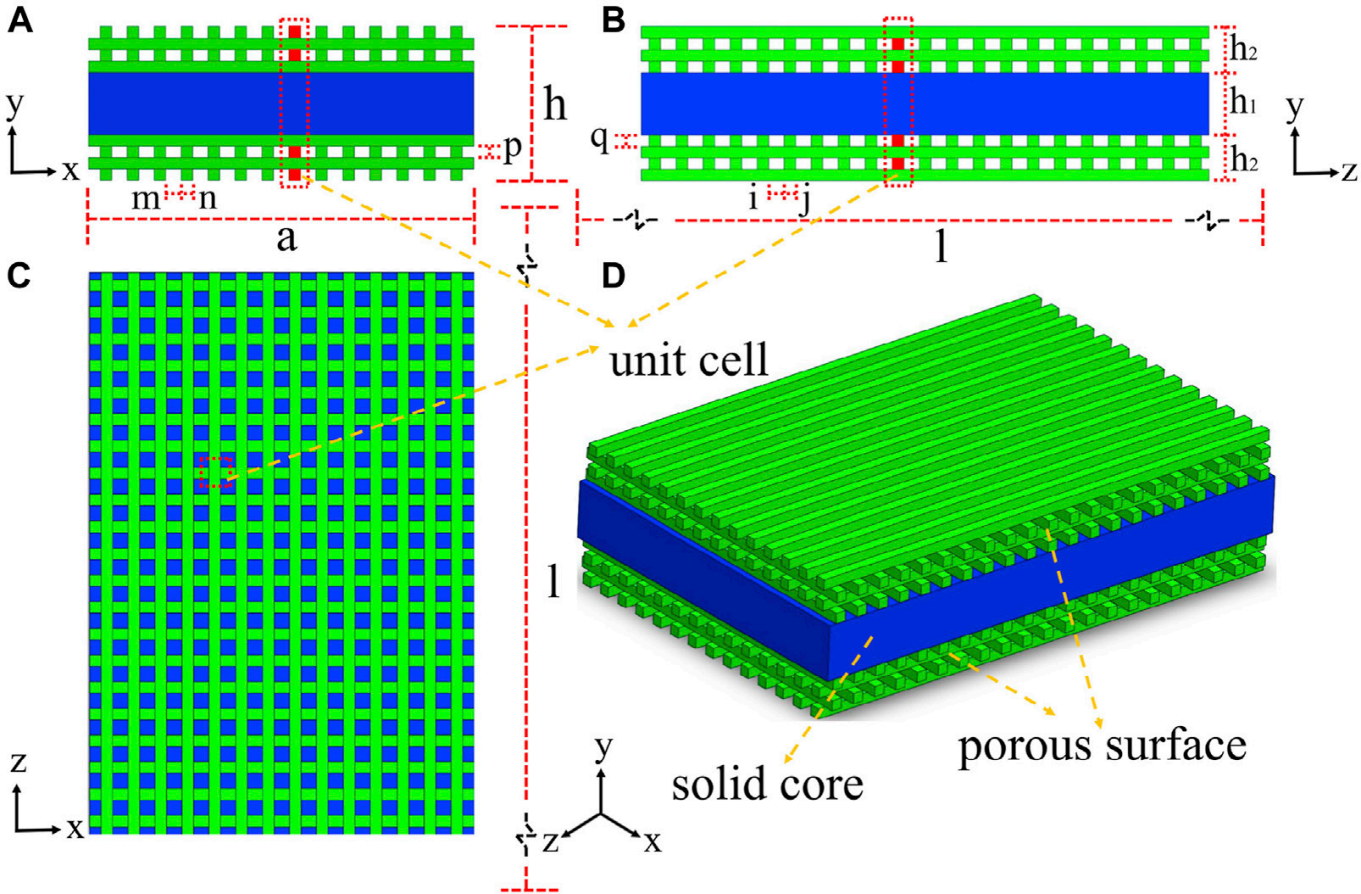
Surface porous PEEK structure with a solid interior by FDM. **(A)** Left view. **(B)** Front view. **(C)** Top view. **(D)** Isometric view.

The unique porous structure on the surface of the 3D-printed scaffold provides high roughness and a large usable surface area, thereby increasing the accumulation of cells in these grooves, which may promote intercellular contact and improve cell viability (Yu et al., 2022). However, the internal pore of the implant reduces its mechanical properties. To balance the mechanical and osseointegration properties of PEEK, Li et al. (2021) designed a surface porous PEEK (SP-PEEK) structure with a solid interior by FDM (Figure 3). It has a variable porous layer number and pore diameter (m = 0.4 mm, 0.6 mm, 0.8 mm, and 1.0 mm). When the pore layer number varied from 2 to 8, SP-PEEK retained the modulus of solid PEEK from 96.78% to 45.59%. When the pore diameter varied from 0.4 to 1.0 mm, SP-PEEK retained the modulus of solid PEEK from 91.58% to 48.00%. In addition to retaining most of the mechanical properties of PEEK, SP-PEEK also exhibited excellent *in vitro* osteogenic behavior. The authors found that the group with m = 0.6 mm had the highest osteogenic activity (*p* ≤ 0.05). It is reported that printing parameters such as nozzle temperature, plate temperature, layer thickness, printing speed, infill ratio, and raster angle also affect the mechanical properties of a 3D-printed material significantly (Wang et al., 2020). Therefore, changing the printing parameters is one of the methods to solve the problem of insufficient mechanical properties of materials. The optimal set of printing parameters for achieving the best mechanical performance has not yet been found. Wang et al. (2021c) designed a three-factor experiment based on the Box–Behnken design and used the response surface methodology (RSM) to find the optimal printing parameters. Among the parameters involved in the experiment, the nozzle diameter had the greatest impact on the mechanical properties of 3D-printed material, followed by printing speed and nozzle temperature. In addition, the authors also proposed a set of parameters that are meaningful for the application of dental implants: the parameter combination of a nozzle diameter of 0.5 mm, a nozzle temperature of 420 C, and a printing speed of 5 mm/s tends to form the best bending strength and elastic modulus simultaneously. A compression test showed that the larger the nozzle diameter (>0.6 mm), the better the compression performance of the 3D-printed material. Sonaye et al. (2022) also found a set of suitable processing conditions to produce FFF-based PEEK with excellent mechanical properties: bedplate temperature 150°C, nozzle temperature 450°C, chamber temperature 90°C, layer thickness 0.1 mm, and printing speed 30 mm/s. They also used an autoclave at 134°C for 15 h (134°C for 1 h in the autoclave is equivalent to 37°C for 1–4 years) to conduct an accelerated aging test. The average fatigue strength of aged PEEK and non-aged PEEK is 27.86 MPa and 32.09 MPa, respectively, so they both can withstand force greater than the maximum oral masticatory force (306 N). This study breaks through the shortcoming of FFF in manufacturing small but robust implants and demonstrates their long-term mechanical durability. In terms of PEEK composites, both printing temperature and composite content can affect the mechanical properties of the printed product. Wang et al. (2021b) found that the tensile and flexural strengths of 5 wt% of CF-reinforced PEEK (CFR-PEEK) in FDM increased with the rise in nozzle and platform temperatures. However, as the introduction of fibers increased, the impact strength of PEEK composites decreased. The aforementioned phenomenon can be explained as the printing material has better melt flow and formability at higher temperatures. In addition, higher temperature provides more energy to increase penetration and diffusion between the filaments and interlayer. The increase in fiber content leads to the formation of pores and the degradation of molecular chain properties during filament preparation. However, temperature requirements for the chamber, print bed, and hot end of FDM-printed PEEK are higher than those for most available commercial FFF printers. Rodzeń et al. (2021b) successfully printed PEEK/hydroxyapatite (PEEK/HA) composites (up to 30 wt% HA) by using a custom-modified commercial printer Ultimaker 2+ (UM2+) with high-temperature capabilities. X-ray diffraction (XRD) showed crystallinity up to 50%, and crystalline domains can be clearly observed using a scanning electron microscope (SEM) and by high-resolution transmission electron microscopy (HR-TEM) analyses. Such high crystallinity significantly enhanced the mechanical properties of 3D-printed samples through delivering continuous crystalline domains in all directions.

### 3.2 Strategies for increasing interlayer adhesion

Compared to injection molding and milling, molecular chains between layers of FDM-based PEEK are less crosslinked and entangled, leading to only small interlayer forces (Guo et al., 2022a). In addition, PEEK has a large melt viscosity and a high melting temperature, which makes interlaminar interface cracking or delamination become one of the most common failure types during clinical use (Lv et al., 2022). Therefore, increasing the fluidity is a key step in improving interlayer adhesion. Nevertheless, the raw materials for FDM are usually PEEK–fiber composites, which are added to enhance the mechanical properties of the scaffold. Rigid fibers such as glass fibers have high hardness, coupled with nozzle size limitations, resulting in an orientation distribution parallel to the interlayer interface rather than a directional distribution, making it impossible to bridge the indirect interface of the printed layer. In addition, adding fibers can further reduce the fluidity of the slurry and reduce interlayer adhesion (Lv et al., 2022). However, if flexible fibers are added, interlayer bridging can be achieved. Adding inorganic fullerene tungsten sulfide (IF-WS) nanoparticles during the production of the fusion filament can reduce the melt viscosity of the polymer by 25% (Golbang et al., 2020). Similar to the layer-by-layer laying process of FDM, Wu et al. (2022) generated CFR-PEEK laminates via laser-assisted forming with a repass treatment. Because of the repass treatment on the top surface of the laminate through laser heating and roller compaction, percolation flow and squeeze flow of the resin and reheating of the laminate body were generated. The interlaminar shear strength (ILSS) of laminates, which had less voids and a higher degree of crystallinity, was improved by 32.87% more than that of the laminates without repass treatment. Post-heat treatment is also suitable for increasing the interlayer mechanical strength of FDM 3D-printed PEEK composites by increasing crystallinity and interfacial bonding properties. Treatment at 250°C for 6 h reduces inter-fiber drawbacks and at 230°C potentially increases the interlaminar tensile strength from 6.96 MPa to 36.28 MPa (Rodzeń et al., 2021a). Since printing parameters can improve the mechanical properties of 3D-printed PEEK, perhaps its mechanism is to increase interlayer adhesion. Basgul et al. (2021) developed a one-dimensional (1D) transient heat transfer-based non-isothermal polymer healing model to predict the interlayer strength of FFF-based PEEK. According to the model, they found an association between nozzle temperature, bed temperature, and environment temperature (TN, TB, and TC, respectively) and the interlayer strength in FDM. The most significant impact on interlayer healing was TN. Decreasing the TN by 20°C–465°C almost halved completely healed layers (47% less), and below 445°C (TN), none of the layers could achieve 100% healing. Increasing TB could increase the number of healing layers by 100%. Although TC had little effect on the lower area near the printing bed, it increased the number of 100% healed layers by heating up. In order to solve the layer delamination and the mechanical performance shortage caused by FDM-based 3D printing and promote its deeper and wider development in the medical field, we should continuously try other methods such as proper material formulation, improving printing parameters, improving compactness of the layer interface and adjustment of the printing interval, and optimization of the printing path (Lv et al., 2022).

Although 3D printing technology has been able to produce customized scaffolds with a complex intrinsic porous structure and different surface roughness, its high cost and low productivity limit its application in large-scale production. The resolution achievable to date also poses a challenge when applying 3D printing to dental implants with a diameter less than 5 mm (He et al., 2021). More importantly, the impact of AM technology and the post-treatment process on crystallinity is difficult to control. Low crystallinity may be due to the insufficient mechanical strength of the material, while high crystallinity can cause deformation of the material (Yi et al., 2021). In the future, we should focus on finding a new manufacturing method that simultaneously improves stiffness and ductility that 3D printing cannot achieve. A solid-state pressure-induced flow (PIF) process uses a mold to apply pressure to a solid material and forces the sample to flow in one direction within the confinement of both sides. PIF can prepare a bioinspired nacre-like PEEK material which has high stiffness and excellent ductility at the same time (Luo et al., 2023).

## 4 Modification for enhanced bioproperties

The aromatic main chain and lack of surface charge and chemical functional groups make pure PEEK exhibit hydrophobicity, low surface energy, and biological inertness. Poor adhesion and proliferation of cells, as well as weak absorption of protein on such an inset surface, lead to reduced osteogenic differentiation of progenitor cells and the production of inflammatory environments which tend to generate apoptosis and necrosis. Finally, fibrous tissue wrapping the implant hinders bone integration, which manifests implantation failure (Olivares-Navarrete et al., 2015; Wan et al., 2020). In recent years, scholars have spared no effort in researching PEEK modification and made it a hot topic, especially for the methods of comprehensive biological response related to bone reconstruction after implantation, including biocompatibility, bacterial resistance, immunoregulation, angiogenesis, antioxidation, osteogenesis and anti-osteoclastogenesis, and soft tissue adhesion. Biocompatibility of dental implants is the basis for affecting protein adsorption and osteoblast adhesion and differentiation. However, insufficient antibacterial activity of the material can lead to the formation of dental plaque on its surface, which, in turn, reduces the biocompatibility of the material surface (Renvert et al., 2008). In addition, bacterial infections around implants can cause bone resorption. Materials prevent inflammation by regulating the body’s immune response, thereby affecting bone remodeling and absorption (Takayanagi, 2007). The unique periodontal soft tissue sealing of dental implants is the first line of defense against external stimuli. During bone regeneration around the implant, it is essential to generate blood vessels. Vascular regeneration ability and blood supply ensure sufficient nutrient supply during osseointegration, which is also a prerequisite for the formation of osseointegration by pre-osteoblasts and mesenchymal stem cells (Ding et al., 2021). It is worth noting that early inflammation caused by implants is beneficial for early angiogenesis and tissue regeneration, while subsequent controlled inflammation can promote bone regeneration (Kargozar et al., 2020). This section comprehensively analyzes and discusses innovative modification methods to achieve separate or simultaneous enhancement of the aforementioned activities, promoting the feasibility and long-term stability of PEEK application in human oral environment.

### 4.1 Biomechanical property

**FIGURE 4:**
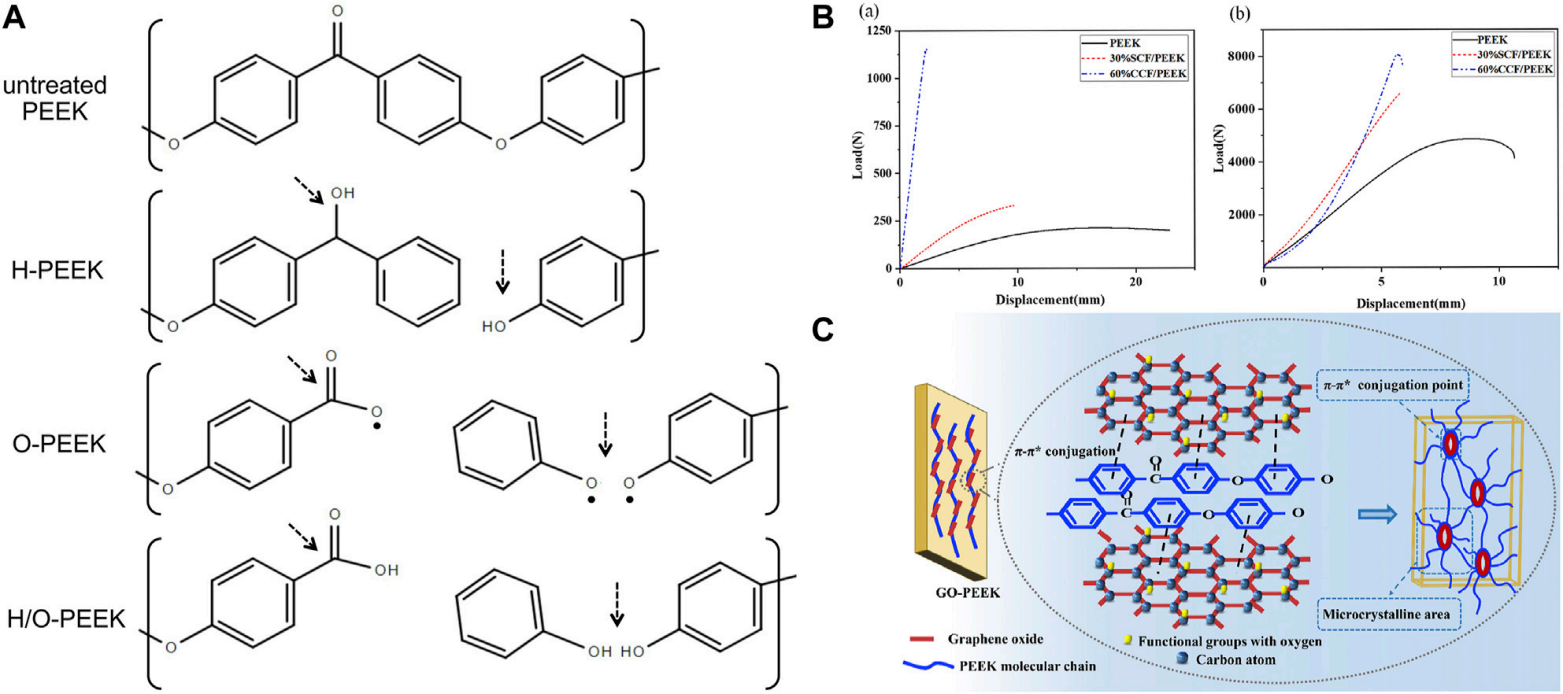
**(A)** Changes in PEEK chemical bonds after different plasma treatments (Fu et al., 2021a). **(B)** Bending (a) and tensile (b) strength tests of 30 wt% short CF-reinforced PEEK and 60 wt% continuous CF-reinforced PEEK (Zhou et al., 2022). **(C)** π–π* conjugations between PEEK and GO (He et al., 2019).

Plasma is an ionized gas with an equal density of positive and negative charges, commonly known as the fourth state substance. They exist in a high-energy state, including electrons, ions, free radicals, and excited species (Wang et al., 2019). Plasma immersion ion implantation (PIII) treatment usually uses high-voltage electricity to accelerate plasma particles and implant them onto the surface of the materials. Such bombardment of the surface can locally heat up at the nano-scale and activate chemical reactions (Comyn et al., 1996). At a more detailed molecular level, strike not only damages the polymer chains on the surface of materials but also leads to microetching, removal of organic residues, and cross-linking. Differences in functional groups and activities introduced by various plasmas attract researchers’ attention. Fu et al. (2021b) found that oxygen plasma-treated PEEK took 5 min to reduce the contact angle of PEEK to 3°, while hydrogen/oxygen-treated PEEK only took 1 min, indicating that H/O plasma worked fastest to achieve the same effects. The author believed it was related to the differences in fracture sites and reformation of functional groups (Figure 4A). During hydrogen plasma treatment, the C–O–C bond and C=O bond of PEEK fractured to form C–OH, and there was a small amount of benzene rings cleaved and volatilized, which were the reasons for hydrophilicity improvement. In oxygen plasma treatment, after the cleavage of C–O–C bonds, an O atom/radical was added to form C–O–O–C, and a benzene ring and the C=O group broke to form unstable O=C–O• and C–O• which reacted with the humidity of air to form O=C–OH and C–OH finally. The generation of these functional polar groups resulted in a smaller contact angle for oxygen plasma treatment compared to hydrogen plasma treatment. The H/O-PEEK group combined the advantages of the aforementioned two types of plasma to produce the fastest working speed. However, the mixture of hydrogen and oxygen was theoretically explosive. The low pressure under study and the safety valves in the system perfectly ensured safety. However, the study did not include the gold implant material standard, Ti, as a control. Other research studies found that ammonia or N_2_ plasma treatment produces nitrogen-containing functional groups, while water plasma treatment produces OH groups (Yu et al., 2022). Although plasma treatment has been proven to optimize the properties of PEEK, the time taken for plasma treatment to generate maximum surface crystallinity, whether it can resist implantation process wear by increasing surface hardness, and the role of bone integration *in vivo* are still a significant focus of future research (Delgado-Ruiz and Romanos, 2018).

In recent years, many studies have focused on synthesizing composite materials of PEEK and fillers. Fillers dispersed in PEEK matrices can significantly improve their mechanical properties, such as elastic modulus, bending strength, and micro-hardness (Zhong et al., 2021). The type and content of fillers determine the characteristics of composite materials. Due to high mechanical performance and low density, carbon fiber (CF) became the most commonly used reinforcing fiber for PEEK (Chua et al., 2021). Different length, thickness, and weight fractions of CF added to PEEK can produce CFR-PEEK with different elastic moduli, which are within the elastic modulus range of human bones (Guo et al., 2022c). Zhou et al. (2022) proposed that the bending and tensile strength and elastic modulus of 30 wt% short CF-reinforced PEEK were close to those of human bones, while the bending strength of 60 wt% continuous CF-reinforced PEEK was 644 MPa (Figure 4B), which is even higher than that of pure Ti. Therefore, such materials with high strength and appropriate elastic modulus are suitable for use as oral implants. The type of CF should be selected based on the application and process. Short CF increases the wear resistance of PEEK but reduces ductility (Ji et al., 2020). Long CFs have strong mechanical properties and can be braided in various ways (Zhao et al., 2021). Continuous CF has the best performance and low cost, but its processing efficiency is low (Chang et al., 2020). However, the cytotoxicity of CFR-PEEK is controversial. Some studies have found that CFR-PEEK has mild cytotoxicity and increases with the increase in CF content (Qin et al., 2019). The differences in manufacturing technology may be the reason for the inconsistent cytotoxicity of CFR-PEEK; therefore, more *in vivo* experiments are needed to determine the biocompatibility of CFR-PEEK.

It is worth noting that when PEEK is reinforced or functionally modified, its mechanical strengths, such as stiffness, tensile strength, flexural strength, and hardness, increase, but its toughness is difficult to balance. Researchers usually functionalize fillers on the surface to evenly disperse or increase interfacial bonding strength in the PEEK matrix, which can promote load transfer but may confine the motion of interfacial polymer segments and lead to a substantial decrease in ductility (He et al., 2019). Graphene (G) and graphene oxide (GO) are low-dimensional nanomaterials which are widely used as reinforcing fillers for polymers. He et al. (2021) manufactured GO-reinforced PEEK (GO/PEEK) nanocomposites with different GO loading through injection molding. The compressive modulus of all composites reached a level similar to that of natural cancellous bone. The 0.5% GO loading had the maximum increase in elongation at break (increased by 86.32% compared to PEEK) and remarkable toughness (increased by 127.20% compared to PEEK). The adhesion and spreading of bone marrow stromal stem cells were also enhanced by the addition of GO. This might be attributed to the structural similarity between PEEK and GO, which enabled them to achieve strong interaction through the formation of π–π* conjugations (Figure 4C). They provided uniform dispersion of GO in the PEEK matrix, nucleation sites for the oriented crystallization zone of PEEK, and increased molecular chain alignment along the GO plane.

### 4.2 Antibacterial property

Currently, about 20% failed implantation surgeries are caused by infections (Deng et al., 2017). Collagen fiber degradation and marginal bone resorption in peri-implantitis are caused by host overreaction and the direct action of bacteria (Abranches et al., 2018). Therefore, the influence of PEEK as a dental implant material on bacterial adhesion and biofilm formation as well as its bactericidal ability is very important. Although many studies have shown that PEEK has an excellent antibacterial rate of about 50%, which is better than that of Ti, bacteria still adhere to the surface of PEEK under SEM observation, and the number increases over time. Therefore, the antibacterial performance of PEEK itself is not sufficient to resist infection, which can cause inflammatory fibrous tissue to wrap around PEEK and hinder bone integration (Najeeb et al., 2016a), affecting the stability and functional load of the dental implant. The following introduces novel antibacterial modification methods of PEEK.

**FIGURE 5:**
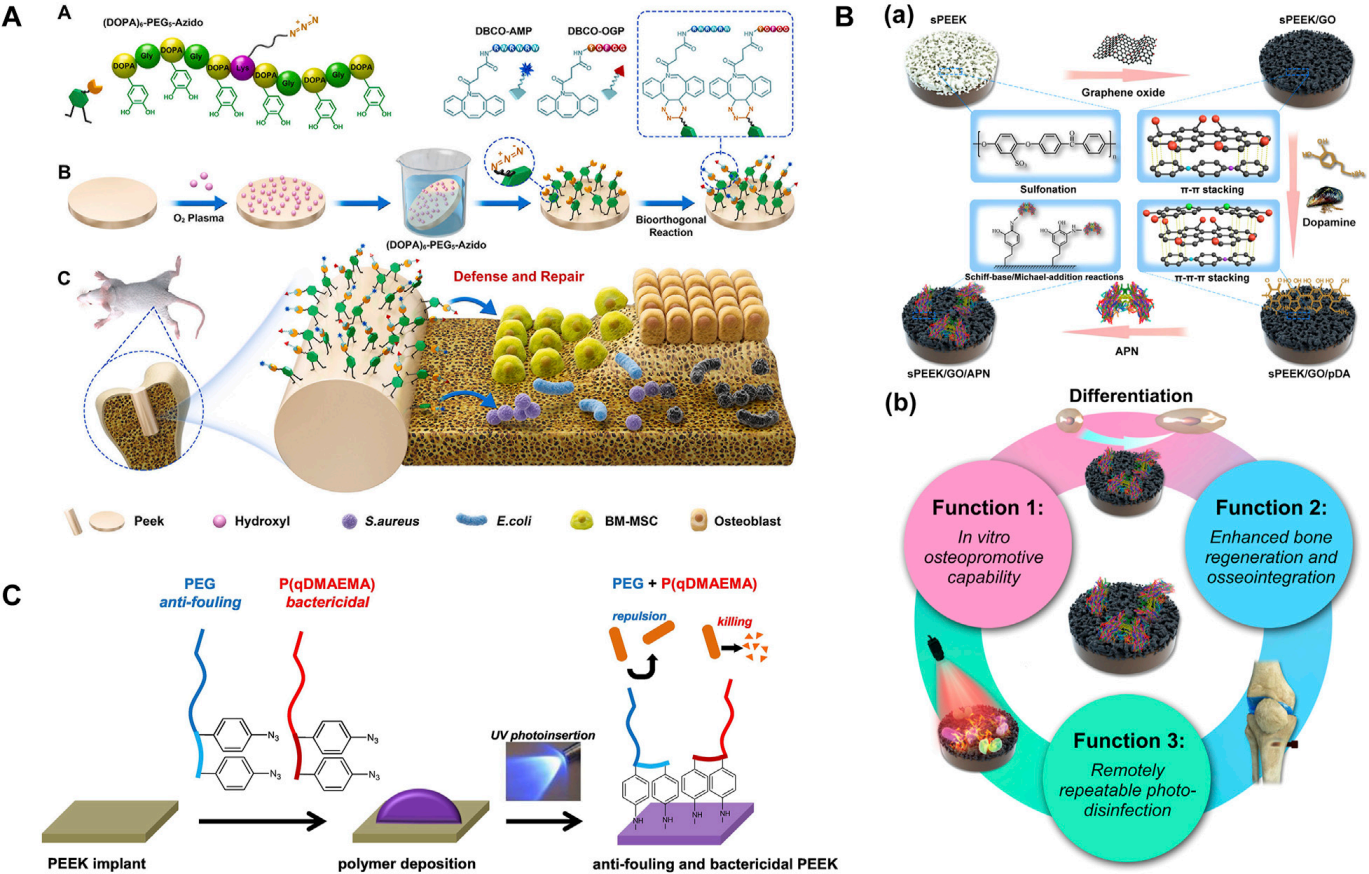
**(A)** PEEK was soaked in a mussel foot protein (Mfp)-mimic peptide with a clickable azido terminal and bio-orthogonally clicked AMP and OGP on azide-modified PEEK in different feeding molar ratios to achieve dual functions of antibacterial property and repair. **(B)** (a) The coating consists of a PDA nanolayer, GO nanosheets, and APN protein. (b) Verification of triple activity (Deng et al., 2020). **(C)** Mechanism of UV photoinsertion (Buwalda et al., 2020).

Subgingival plaque is composed of *Streptococcus sanguinis* (*S. sanguinis*) and *Porphyromonas gingivalis* (*P. gingivalis*) and is the initiating factor of peri-implantitis. They are the early and late colonized bacteria in the dental plaque biofilm, respectively (Periasamy and Kolenbrander, 2010). Some metal cations can inhibit pathogenic bacteria of peri-implantitis, such as silver (Ag^+^), zinc (Zn^2+^), magnesium (Mg^2+^), and copper (Cu^2+^) ions. However, the potential toxicity of metal ions and the antibiotic resistance mutations of bacteria no longer make them perfect antibacterial agents, and people are increasingly in urgent need of antibacterial agents with strong antibacterial effects, few side effects, and no drug resistance. Efficient and aggressive antimicrobial peptides (AMPs) have become a new kind of antibacterial agents because of their biogenic nature (Jiang et al., 2021). AMPs not only exert bactericidal effects by targeting bacterial cytoplasmic membranes and dislocating the adhesion of mussel-like molecules to PEEK but also left azide groups that could undergo orthogonal reactions (Wenzel et al., 2014; Chen et al., 2020a). Li et al. (2022a) bio-orthogonally clicked AMP and an osteogenic growth peptide (OGP) on azide-modified PEEK (DBCO-AMP and DBCO-OGP) in different and accurate feeding molar ratios to achieve dual functions of defense and repair (Figure 5A). *In vitro* and *in vivo* experiments had shown that the AMP-containing group could degrade *Escherichia coli* (*E. coli*) and *Staphylococcus aureus* (*S. aureus*) and integrated better with surrounding tissues, even synergistically enhancing bone integration in the case of postoperative infection. The study drew heatmaps with various standardized performances and found that PEEK–A_2_O_2_ (the feeding molar ratio of AMP/OGP was 2:2) had the best dual activity. Due to the limited active site on the surface of PEEK, antibacterial and osteogenic modifications are contradictory in most cases (Yakufu et al., 2020), so it is important to find a balance between them. Simply increasing the content of osteogenic-inducing active substances does not significantly enhance the bone integration effect *in vivo* as antibacterial activity is a prerequisite for osteogenesis. This study may be a promising solution in the field of surface bioengineering modification of inert dental implants. Yuan et al. (2019) decorated mouse beta-defensin-14 (MBD-14) on porous PEEK via lyophilization, and the modified PEEK was verified to have broad-spectrum antibacterial ability through *in vitro* and *in vivo* experiments. Proliferation and osteogenic differentiation of bone mesenchymal stem cells were also enhanced. Although antibiotics are one of the most commonly used antibacterial agents, one in every 15 people is allergic. Furthermore, the form of dental bacterial biofilm has antibiotic resistance which is 1,000–1,500 times greater than that of planktonic bacteria. Photodynamic therapy (PDT) is a light-based alternative therapy for peri-implantitis and peri-implant mucositis, especially when patients are allergic to antibiotics (Sibata et al., 2000). PDT uses photosensitizers that can be activated when exposed to specific wavelengths of light in the presence of oxygen (typically using visible red light at 620–690 nm). Focusing the light on the infected lesion, the photosensitizer transfers energy to oxygen molecules, converting them into strongly oxidizing singlet oxygen. Ultimately, the production of reactive oxygen species (ROS) leads to bacterial death (Robertson et al., 2009; Azizi et al., 2018; Ren et al., 2020b; Hou et al., 2020). Peng et al. (2021a) compared the antibacterial effects of PDT and ampicillin through biofilm removal assay. Different concentrations of temoporfin were selected as photosensitizers. The results showed that both the PDT and ampicillin groups had good removal effects on *Streptococcus mutans* (*S. mutans*) and actinomycetes (*p* < 0.05), with high doses of temoporfin having better biofilm removal effects (*p* < 0.01). The osteoblast activity of PEEK was comparable to that of other groups. Deng et al. (2020) prepared a coating consisting of a PDA nanolayer, GO nanosheets, and adiponectin (APN) protein on the surface of sulfonated PEEK using π–π interaction (Figure 5B). After irradiation with 808-nm NIR light, the coated PEEK produced antibacterial rates of 99.49% and 92.4% for *S. aureus* and *E. coli,* respectively. However, the damage of high temperature and ROS to surrounding tissues and cells limits its clinical application (Ren et al., 2020b). In the future, how to improve the absorption rate and penetration ability of photosensitizers and develop light sources that can reduce irradiation time are urgent issues that need to be solved.

It is reasonable to achieve antibacterial performance by changing the PEEK surface (morphology and chemical composition). The rigid cell walls of bacteria limit their deformability. When the nanopore size of the porous morphology formed on the surface of PEEK is smaller than bacteria, they cannot stick, while combining other modification methods can kill bacteria with smaller pore sizes. In addition, nanoprotrusion structures can damage bacterial cell membranes (Wang et al., 2017; Wang et al., 2018; Yang et al., 2021). In order to improve the competitive adhesion of cells against bacteria, it is important to comprehensively consider the structure and morphology of bacteria and cells, as well as the properties of materials. Changes in chemical composition usually refer to the combination of PEEK with antibacterial chemicals. The diphenylketone groups on the PEEK main chain are converted to semi-benzopinacol radicals under ultraviolet (UV) irradiation, and then, antibacterial monomers can be grafted spontaneously onto the free radicals (Kyomoto and Ishihara, 2009). There are usually two methods for endowing PEEK with antibacterial activity: (1) reducing bacterial adhesion and (2) killing bacteria. Buwalda et al. (2020) achieved the aforementioned two aspects simultaneously through a one-step approach. They covalently grafted bactericidal quaternized poly(dimethylaminoethyl acrylate) (P (qDMAEMA)) polymers and aryl–azide-containing modified anti-fouling PEG onto the surface of PEEK through via UV photoinsertion (Figure 5C). Although such functionalized PEEK had no obvious effect on Gram-negative *E. coli*, it had a good inhibitory effect on Gram-positive *S. aureus*, which increased with PEG chain length. It is worth noting that only when the lengths of PEG and P (qDMAEMA) were roughly equal could they exert a synergistic effect. The method of inserting aryl–azide groups through UV photoinsertion had “spectral significance” because most polymer substrates with carbon–hydrogen bonds could form covalent bonds with reactive nitrene intermediates. Therefore, this chemical method can be extended to other polymer implants. Positive antibacterial groups, such as -SO_3_H, -OH, and -COOH, can also be introduced on the surface of PEEK, which can cause electrostatic repulsion and negatively charged bacterial cell membranes to generate a zeta potential difference (Liu et al., 2021a; Zhang et al., 2022). However, the introduction of these functional groups lacks long-term *in vivo* experimental verification.

In addition to changing the surface properties of PEEK, blending modification can enhance its antibacterial activity. Studies have confirmed that functionalized ceramic nanoparticles such as titanium dioxide (T-NPs) and silicon dioxide (S-NPs) had antibacterial activity under UV and dark conditions (Bokare et al., 2013). In the absence of UV irradiation, ceramic particles can produce exogenous ROS to exert bactericidal effects (Díez-Pascual and Díez-Vicente, 2015). Muthusamy Subramanian and Thanigachalam, (2022) used T-NP- and S-NP-reinforced PEEK (T/PEEK and S/PEEK) with good compressive strength and hardness values to test antibacterial activity *in vitro*. The average diameter of the inhibitory zone of 16 wt% T/PEEK, 12 wt% S/PEEK, and 16 wt% TS/PEEK on *E. coli* was 10.5, 11.9, and 18.299 mm, respectively, and the average diameter of the inhibitory zone on *Bacillus subtilis* was 12.25, 13.65, and 16.125 mm, respectively. The inhibitory zone diameters of pure PEEK were 9.213 mm and 10.452 mm, respectively. Therefore, it was confirmed that even without UV irradiation, the antibacterial ability of T-NP- and S-NP-reinforced PEEK composites can be significantly improved. Pezzotti et al. (2018) mixed silicon nitride (Si_3_N_4_) of three phases and PEEK by high-temperature melting. *In vitro* experiments found that PEEK/β-Si_3_N_4_ had the best antibacterial effect against *Staphylococcus epidermidis* (*S. epidermidis*), while there was no obvious difference in the performance of PEEK/α-Si_3_N_4_ compared to pure PEEK. The authors explained that the eluted NH^3+^ increased the pH value around the implant and damaged the bacterial cell membrane. Although antibacterial fillers can avoid the uncertain long-term bonding stability of coatings, they can alter the overall mechanical properties of the composite material. In addition, there is also a problem of weak bonding between the filler and PEEK interface. It is necessary to evaluate the functional loading of PEEK composite materials after implantation *in vivo*, and finding suitable filler dosage and size is also a key research point.

Most modification strategies of PEEK are to increase its activity by improving hydrophilicity, but as hydrophilicity increases, bacterial adhesion to its surface increases subsequently. Lijnev et al. (2022) incubated PEEK in 10 M sodium hydroxide for 24 h at 37°C. As expected, the enriched -OH increased the hydrophilicity of PEEK, thereby increasing its protein adsorption, mineral deposition, and human bone mesenchymal stem cell (hBMSC) adhesion. However, the disc diffusion method and *in vitro* bacterial attachment assay results showed a surprisingly significant decrease in the antibacterial activity of PEEK against *S. aureus* strains after the coating treatment. Thus, in order to find better ways to improve the antibacterial performance of dental implants, it is necessary to first understand the mechanism of infection, then evaluate the overall performance of the modification method, and finally, conduct animal and clinical experiments.

### 4.3 Immunoregulation

Inflammation begins with bacterial infection, followed by an excessive immune response mediated by autoimmune cells. When dental implants are screwed into the maxilla and (or) mandible, macrophages play a core regulatory role in mediating the immune response of the host and releasing cytokines and growth factors, leading to the formation of a pro-inflammatory M1 phenotype or anti-inflammatory M2 phenotype through polarization (Brown et al., 2012; Klopfleisch, 2016). The M1 phenotype enhances inflammatory response in the early stage to control infection, while the M2 phenotype promotes tissue regeneration and repair in the later stage (Shen et al., 2021). For the surface of PEEK, macrophages typically polarize toward the pro-inflammatory M1 phenotype and fuse into multinucleated giant cells, releasing fibrosis-enhancing cytokines, ultimately blocking osseointegration by fiber encapsulation (Sridharan et al., 2015). Therefore, PEEK is extremely desirable to be endowed with immunomodulatory ability, enabling it to transition from transient M1 polarization to an anti-inflammatory M2 phenotype in a timely manner, which can release chemokines to recruit osteoprogenitor cells and activate osseointegration (Chen et al., 2016). Based on the aforementioned mechanism, some studies loaded interleukin-4 (IL-4) on the implant surface to polarize macrophages to the M2 phenotype (Spiller et al., 2015) or used covalent modification of clusters of differentiation 47 (CD47) protein to “camouflage” which could not be identified by the autoimmune system (Gao et al., 2017). However, these expensive, short-life, and complex preparation processes of growth factor proteins are not easy to come by. Enabling dental implant materials to induce M2 phenotype macrophage polarization and creating a suitable osteogenic microenvironment have attracted extensive interest recently.

**FIGURE 6:**
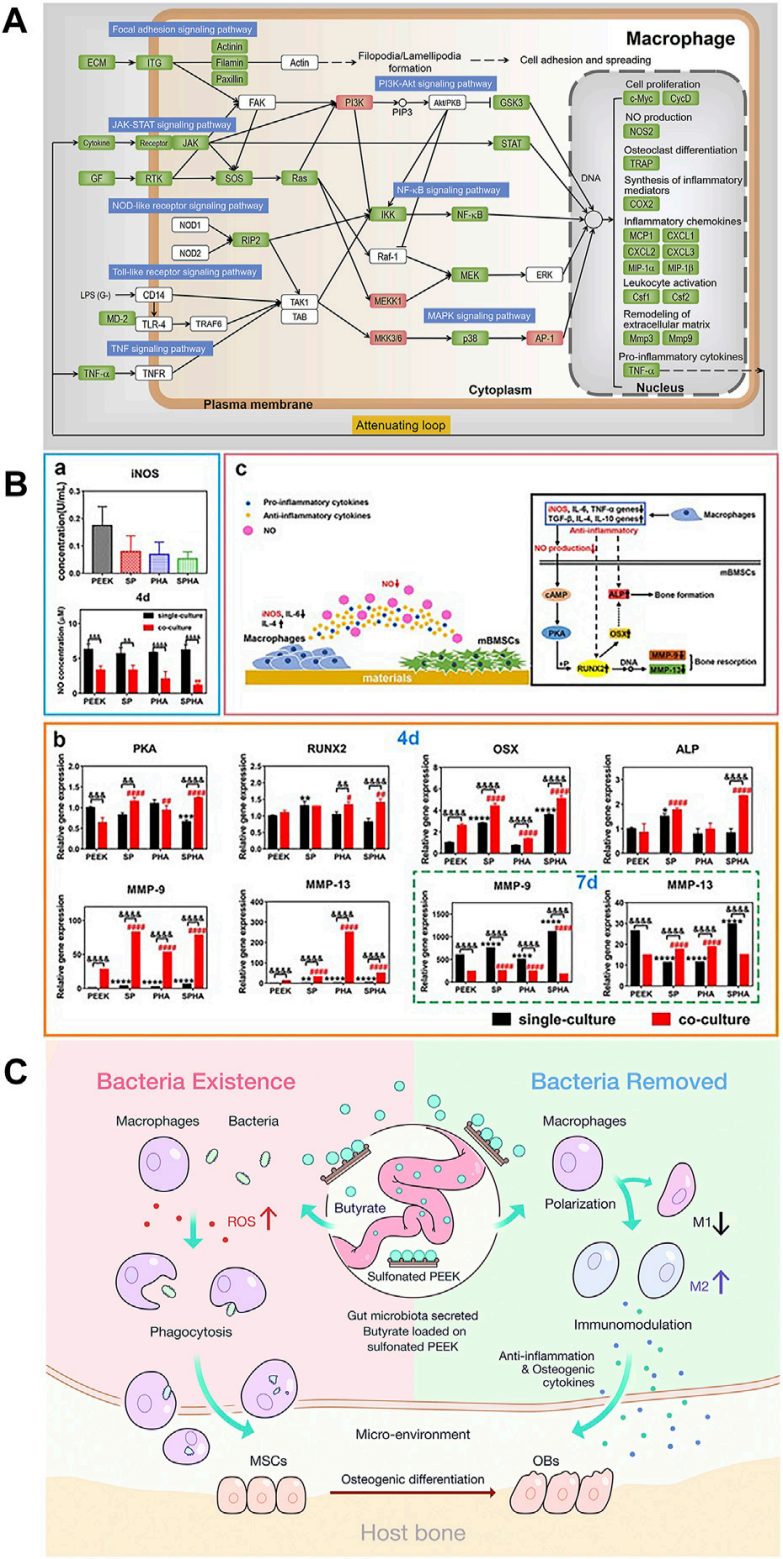
**(A)** Key signaling transduction cascades induced by pH 1.8 (Gao et al., 2020). **(B)** Concentration of iNOs and NO. (b) Expression of osteogenic-related genes. (c) Interaction of materials, macrophages, and mBMSCs (Liu et al., 2022). **(C)** Under the presence of bacteria, the production of ROS increases SB content and macrophage activity. SB mainly induces macrophage polarization toward M2 and promotes osteogenesis (Yue et al., 2018).

As a cost-effective technology, layer-by-layer (LBL) self-assembly can form films with specific structure and composition on the surface of materials by continuously dipping in polyelectrolytes with opposite charges (Costa and Mano, 2014). The films formed on the surface of PEEK through electrostatic interactions change its surface morphology, which can not only induce hBMSCs to differentiate into osteogenic lineage but also selectively polarize macrophages to reduce the secretion of pro-inflammatory cytokines, endowing PEEK with immunomodulatory ability (Gao et al., 2020). Gao et al. (2020) repeatedly immersed negatively charged PEEK into 2-mercaptoethanol, phorbol-12-myristate-13-acetate (PAH), and poly(acrylic acid) (PAA) weak polyelectrolyte solutions. The cation NH^4+^ of the PAH chain and the anion COO^−^ of the PAA chain were electrostatically attracted and self-assembled. The study found that the expression of integrin (ITG) subunits (α4, α5, αM, αX, αD, β2, and β7) and adhesion complexes (actinin, filamin, and paxillin) on pH 1.8 was lower, leading to a decrease in focal adhesion of macrophages. The expression of lymphocyte antigen 96 (MD-2) was inhibited, resulting in the negative regulation of the Toll-like receptor (TLR) signaling pathway as well as downstream signaling cascades. The activation of receptor-interacting protein 2 (RIP2) was weakened, leading to a downregulation of the nucleotide-binding and oligomerization domain-like receptor (NLR) signaling pathway. The aforementioned effects ultimately led to a decrease in the transcription of inflammation-related genes, especially the instantaneous activation and secretion of pro-inflammatory cytokines TNF-α (the aforementioned key signaling transduction cascades are shown in Figure 6A) which was confirmed by enzyme-linked immunosorbent assay (ELISA). This meant that pH 1.8 caused macrophages to enter a weakened feedback loop, silencing acute inflammation and upregulating osteogenic-related genes by polarizing to the M2 phenotype.

Excluding coatings, changing the physical and chemical properties of the PEEK surface, such as morphology (Lee et al., 2018), functional group types (Vassey et al., 2020), and elemental composition (Guo et al., 2021b), can endow PEEK with unique characteristics and improve its hydrophilicity. Subsequently, the polarization of adherent immune cells will be directly affected, which regulates the host immune response (Qin et al., 2020). Designing micro/nano-morphological structures of PEEK is one of the most commonly used and valuable strategies for improving immune regulatory ability. Surface physical structure and chemical composition modification of PEEK definitely exhibit better synergistic effects than single modification. Liu et al. (2022) sulfonated PEEK/hydroxyapatite (HA) (SPHA) composites to obtain both 3D porous physical and Ca^2+^ chemical signal surfaces. High concentration of extracellular calcium could reduce inflammation via activating the calcium-sensing receptor signal cascade to inhibit tumor necrosis factor alpha (TNF-α) expression and the wingless type 5a/receptor tyrosine kinase-like orphan receptor 2 (Wnt5a/Ror2) signaling pathway (Chen et al., 2016). Therefore, the high Ca^2+^ concentration and hydrophilicity of sulfonated PEEK/HA resulted in a low M1 phenotype–macrophage ratio. In addition, SPHA downregulated the expression of the inducible nitric oxide synthase (iNOS) protein, resulting in a nitric oxide (NO) concentration decrease (Zhou et al., 2021). In the co-culture medium of SPHA and mouse bone marrow mesenchymal stem cells (mBMSCs), due to low NO concentration, the expression of osteogenic-related osterix (OSX, the downstream gene of runt-related transcription factor 2 (IRUNX2)) and alkaline phosphatase (ALP) genes was increased in 4 days (Figure 6B) through the cyclic adenosine monophosphate–protein kinase A (cAMP-PKA) pathway (Kim et al., 2021), while the expression of osteoclast-related matrix metalloproteinase-9 (MMP-9) and MMP-13 (MMPs degrading the mineralized matrix) genes was reduced in 7 days. Some studies combine porous surfaces with the direct loading of immunomodulatory bioactive molecules or substances. BMSCs play a strong role in osteoimmunomodulation because of BMSC-derived exosomes (Exos) which carry biosignal molecules in paracrine secretion (Li et al., 2020). Exos regulate the transformation of macrophages from the M1 phenotype to M2 phenotype after binding to target cells. In addition, Exos carry a variety of miRNAs related to regulating osteogenesis, which can directly induce internal and external osteogenesis in the absence of cells (Zhai et al., 2020). Fan et al. (2021) bridged BMSC-derived Exos coating onto 3D porous PEEK via tannic acid (TA). RT-PCR and immunofluorescence results showed that the Exo-coated TA-SPEEK group could inhibit the expression of M1 surface markers (TNF-α and iNOS) and promoted the expression of M2 surface markers (Arg-1 and IL-10). The study also found that compared with other groups, the expression of activator phosphorylated IκB (p-IκB) of nuclear factor-kappa B (NF-κB) and the protein phosphorylation degree of downstream factor NF-κB p65 in the Exo-loaded TA-SPEEK group were significantly downregulated, which fully proved that Exo-loaded TA-SPEEK promoted the anti-inflammatory M2 polarization of macrophages through the negative regulation of the NF-κB pathway. Gut microbiota (GM) is crucial in regulating systemic health as its fermentation metabolite short-chain fatty acids (SCFAs) contain butyrate, which is known to have anti-inflammatory and immunomodulatory effects (Geva-Zatorsky et al., 2017). Yue et al. (2018) loaded sodium butyrate (SB) on the surface of SPEEK to study its regulation on macrophages under different stimuli. *In vitro* macrophage polarization assay showed that SB-SPEEK at low concentrations (≤1.0 mM) could increase the expression of M2 phenotype-related cytokines IL-4, IL-10, bone morphogenetic protein-2 (BMP-2), and VEGF (immunomodulatory mechanisms are shown in Figure 6C). However, it is important to pay attention to the potential cytotoxicity hazards of high SB concentrations.

Although there have been many studies on macrophage polarization, the mechanism is too complex and difficult to figure out, including M1/M2 phenotype interconversion, promotion or inhibition of the M1/M2 phenotype, and change in macrophage activity (Murray, 2017). It is worth noting that specific directional polarization M2 phenotype macrophages contain many subtypes, including M2a, M2b, M2c and M2d, and M2a, among which M2c can cause fibrous tissue proliferation (Lescoat et al., 2020). Therefore, in the future, we should find a new regulatory cellular signaling pathway and deeply explore the mechanism of macrophage polarization. Changing the host immune response to materials can determine the fate of dental implants and the outcome of bone integration.

### 4.4 Antioxidative property

Our biological system produces highly active molecules such as ROS and reactive nitrogen species (RNS) during fatty acid metabolism, aerobic metabolism, and when encountering environmental stimuli (Yu and Wang, 2022). The implantation inevitably leads to the release of ROS, such as hydroxyl radical (OH), hydrogen peroxide (H_2_O_2_), and superoxide anion (O_2_^−^), thereby clearing aging cell debris, resisting pathogens, and protecting body homeostasis (Li et al., 2023). The human body’s self-antioxidant defense system can maintain a balance between the oxidative and antioxidative modes by timely removing oxidation–reduction products but lacks the ability to reverse imbalance. Once factors cause imbalance, such as diabetes mellitus (DM), it will cause oxidative stress (OS) in the bone microenvironment around the implants. The excessive ROS produced by OS not only inhibits cell proliferation and differentiation, resulting in tissue damage, but also suppresses the release of VEGF, resulting in impaired capillary formation. In more severe cases, it can damage proteins and DNA (Wang et al., 2021a). Therefore, endowing PEEK dental implants with antioxidative capacity, reducing the impact of OS on nutrient supply, and providing a stable microenvironment for bone integration are as important as osteogenic induction.

**FIGURE 7:**
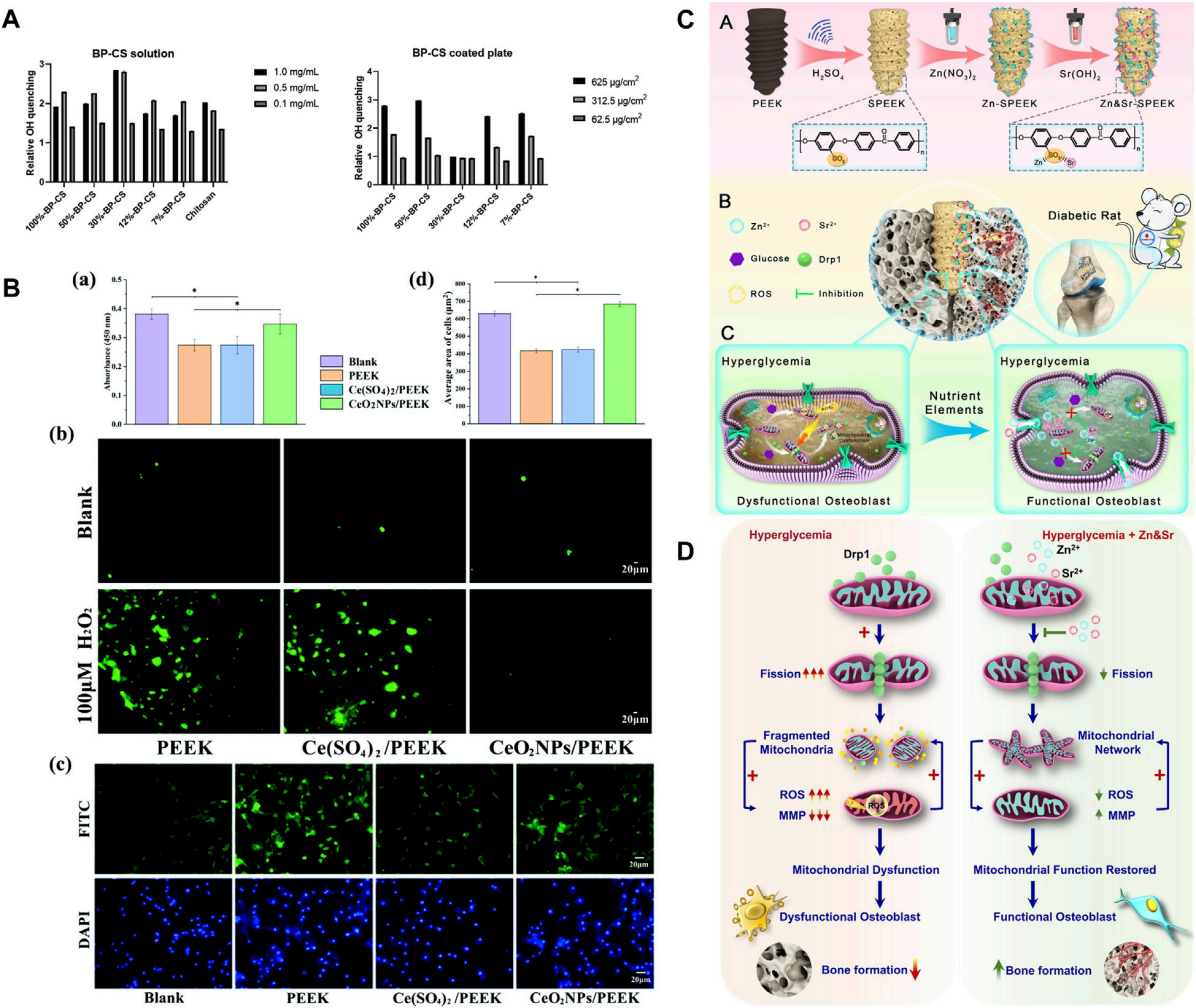
**(A)** Quenching of OH radicals by 30%BP-CS solutions (left) and coatings (right) (Borgolte et al., 2022). **(B)** Under OS conditions: (a) Cell viability, (b) ROS in cells, (c) cell morphology staining, and (d) average cell area (Li et al., 2023). **(C)** Material preparation and *in vivo* bone integration (Wang et al., 2021a). **(D)** Mechanism of tissue damage and targeting mitochondria to promote osteogenesis in DM (Wang et al., 2021b).

Chitosan (CS) has been proven to have antioxidant property that can quench hydroxyl and superoxide radicals (Li et al., 2012). Borgolte et al. (2022) proposed that coating CS with a 30% degree of substitution of benzophenone (30%BP-CS) on PEEK had the best free radical-quenching effect, which could increase the quenching of OH by three times compared to the control group. Furthermore, the quenching effect of 30%BP-CS was 1.5 times higher than that of CS (Figure 7A). The study found that the oxidation resistance of 30%BP-CS coating was not a good solution because the free radical quenching active surface of the coating was relatively smaller. Bone integration includes three synergistic and sequential processes: macrophage-mediated immune response, EC-induced angiogenesis, and osteoblast-induced osteogenesis (Patel et al., 2020). CS can control the multi-stage release of Zn^2+^ and match it with various steps of bone integration. A covalently grafted multifunctional sustained-release coating composed of carboxylated graphene oxide (GC), Zn^2+^, and CS on the surface of CFR-PEEK (CP/GC@Zn/CS) was achieved by Zhao et al. (2023), which completed the rapid release of Zn^2+^ in the initial stage, sustained release of Zn^2+^ in the middle stage, and slow release of Zn^2+^ in the late stage. The coating played a role in immune regulation, angiogenesis, and osteogenesis in stages. The study found that the levels of ROS and RNS in macrophages of CP/GC@Zn/CS were the lowest, thus confirming that the coating can effectively inhibit OS in macrophages. During the removal of H_2_O_2_, cerium dioxide nanoparticles (CeO_2_NPs) have been confirmed to have catalase-like activity and co-act as an oxygen buffer. Li et al. (2023) uniformly doped CeO_2_NPs in PEEK, and its ability to significantly reduce ROS levels in osteoblasts *in vitro* (Figure 7B shows antioxidant capacity tests) and better induce osteogenesis *in vivo* was verified. DM is an extremely common chronic disease, and the mitochondrial dynamics in the microenvironment surrounding the implant are imbalanced due to its sustained hyperglycemia and excessive ROS production (Willems et al., 2015). Therefore, the antioxidant activity of dental implants in DM patients is particularly important. Under high-glucose conditions, dynamin-related protein 1 (Drp1) produces excessive ROS and mitochondrial breakage, while the balance of mitochondrial membrane potential (MMP) resurgence protects the mitochondrial ultrastructure. Based on mitochondrial dynamics and DM osteogenesis, Wang et al. (2021a) loaded ZnO and Sr(OH)_2_ on SPEEK. The study proposed for the first time that the release of Zn and Sr downregulated the DrP1 gene, restored MMP, eliminated ROS, and enhanced bone integration *in vivo* under DM conditions (Figures 7C, D show the mechanisms). Such a development of new PEEK implants targeting the mitochondrial regulatory ability of DM patients is of great significance.

In summary, reducing the degree of intracellular OS through PEEK modification is crucial for creating a favorable bone integration microenvironment. The production of ROS not only directly damages periodontal tissue but also releases chemokines that affect macrophage polarization and activate inflammatory response. Some studies suggest that the reduction of ROS can inhibit the M1 phenotype and promote the M2 phenotype (Yu and Wang, 2022). Therefore, regulating the body immune response by controlling the ROS level is worth further exploration. In addition, antioxidant activity is particularly important in some special patients, such as elderly patients with high OS in the bone microenvironment and DM patients with mitochondrial dynamic imbalance.

### 4.5 Osteogenesis and anti-osteoclastogenesis

Targeting the recruitment of BMSCs around dental implants, promoting cell migration, adhesion, proliferation, and differentiation, and further promoting extracellular matrix (ECM) mineralization are eternal topics and the ultimate goal of dental implant materials. During bone integration, the formation of mineralized bone and the absorption and degradation of the bone matrix are inseparable factors that always work together. The modification of PEEK implants should not only focus on inducing direct osteogenesis or anti-osteoclastogenesis but also on the immune inflammatory response mediated by macrophages that occurs early after implantation. The cross-regulation between the skeletal and immune systems is crucial for the dynamic balance between bone formation and bone resorption which guides successful bone integration (Chen et al., 2016). Therefore, this section introduces PEEK implant modification methods from the perspective of bone immunology that can simultaneously promote osteogenesis and anti-osteoclastogenesis.

**FIGURE 8:**
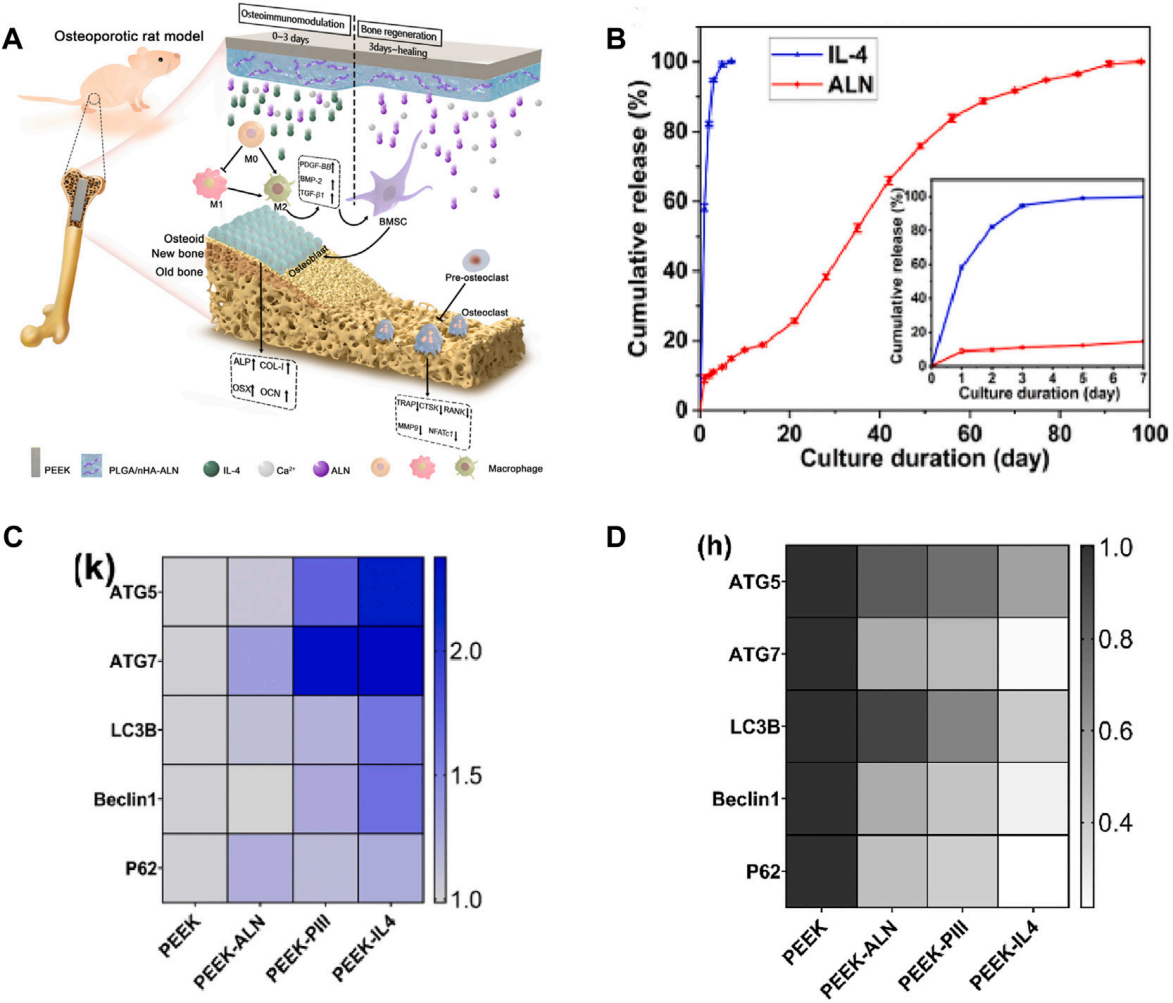
**(A)** Schematic diagram of the programmed regulation of early anti-inflammatory and late osteogenesis. **(B)** *In vitro* release assay. **(C)** Expression of autophagy-related genes after cultivating for 3 days. **(D)** Expression of autophagy-related genes after inducing for 9 days (Zheng et al., 2022).

The unique surface characteristics of implants have a direct impact on the proliferation and differentiation behavior of BMSCs. Yang et al. (2022) constructed a titanate nano-network structure on the PEEK surface (PEEK-TNS) by plasma sputtering and alkali treatment. PEEK-TNS significantly downregulated pro-inflammatory genes (TNF-α and IL-6) and upregulated anti-inflammatory genes (IL-10 and Arg-1), inducing the macrophages transformation from M1 to M2. While the expression of osteogenic-related genes (BMP-6 and OSM) was enhanced, osteoclast-related genes (MSCF and RANKL) were inhibited over time. The loading of osteoporosis (OP) treatment drugs can achieve bone immune regulation of implants. Icariin (ICA) is a natural herbal medicine widely used in the treatment of inflammatory diseases. To avoid the premature release and degradation of ICA, Chai et al. (2022) firmly adhered ICA to SPEEK through PDA (ICA-PDA@SPEEK). The upregulation of BMP-2 and VEGF gene expression in macrophages of ICA-PDA@SPEEK could promote osteogenesis by activating the BMP signaling pathway. Osteoclast-related genes (TRAP, CSTK, and NFATc1) were inhibited. This may be related to the inhibition of the NF-kB signaling pathway (Lecaille et al., 2020). According to reports, enoxacin (ENX) can inhibit RANKL-induced JNK signaling to reduce osteoclast generation. Bai et al. (2023) used polyvinyl butyral (PVB) as the coating medium to modify SPEEK with ENX (SPEEK/PVB-ENX*3). The research found that SPEEK/PVB-ENX*3 can weaken the ability of macrophages co-cultured with the RANKL-inducing factor to fuse into multinucleated osteoclasts. The implantation experiment in rats also demonstrated the function of SPEEK/PVB-ENX*3 in promoting mineralization and osteogenesis. Among the bone resorption inhibitors of OP, sodium alendronate (ALN) is considered to have the best effect. Zhao et al. (2022) doped ALN and Sr^2+^-doped bioactive glasses (SrBGs) into the PEEK matrix. Sr has a dual nature of promoting osteogenic differentiation and osteoclast differentiation. As SrBG gradually degrades, Sr^2+^, Ca^2+^, and ALN exert a synergistic effect. This study observed an increase in the ratio of OGP/RANKL, enhanced osteogenic activity, and inhibition of osteoclasts. Zheng et al. (2022) co-loaded ALN, poly(lactide-co-glycolide) (PLGA), and IL-4 onto the surface of PEEK to programmatically regulate immune response and bone regeneration (Figure 8A). IL-4 rapidly released 90% within 3 days after implantation, promoting the transformation of M1 phenotype macrophages into the M2 phenotype, thereby creating a favorable immune microenvironment. ALN and Ca^2+^ were subsequently released for up to 98 days, promoting bone regeneration and inhibiting bone resorption (Figure 8B). Most interestingly, the study revealed that autophagy, which had immune detection effects, was promoted in the early stages to be anti-inflammatory while being suppressed in the later stages to reduce bone resorption (Figures 8C, D).

Bone integration is a delicate balance between bone resorption and bone regeneration. The improvement in the bone integration ability of PEEK dental implants should involve both osteogenesis and osteoclasis. Osteoclasts are produced by the monocyte macrophage system, so modification strategies targeting the immune system can affect bone remodeling around implants. Such a modification strategy is particularly effective for OP patients as OP typically manifests as overactive osteoclast bone resorption (Zhao et al., 2022).

### 4.6 Angiogenesis

If an appropriate inflammatory response of the implant is a prerequisite for successful osseointegration, then angiogenesis is a concurrent event that assists osseointegration (Kusumbe et al., 2014). Being one of the key factors in osseointegration, angiogenesis is the process of vascular endothelial cell (EC) proliferation in pre-existing vessels to form sufficient blood vessels (Wang et al., 2013). As transportation pipelines, these new blood vessels deliver various nutrients, oxygen, stem cells, and even osteoinductive factors required for the new bone around the implant (Ito et al., 2006; Hutton and Grayson, 2014). However, research studies on PEEK modification methods dedicated to enhancing its angiogenetic ability currently are not as extensive as inducing osteogenic differentiation.

**FIGURE 9:**
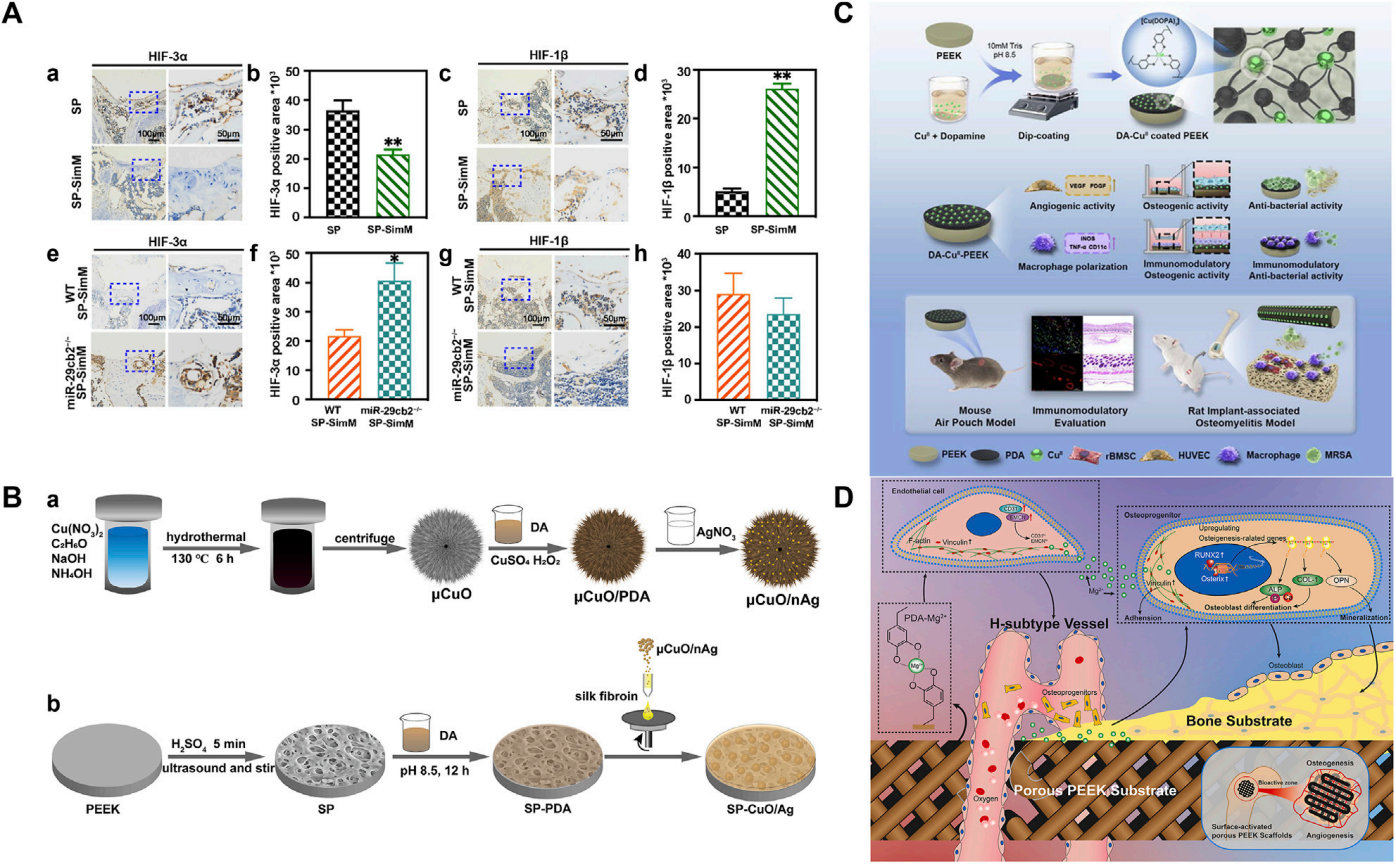
**(A)** Expression and quantification of HIP-3α in wild-type mice (a and b). Expression and quantification of HIF-1β in wild-type mice (c and d). Expression and quantification of HIP-3α in 29cb^2^−/− mice (e and f). Expression and quantification of HIF-1β in 29cb^2^−/− mice (g and h) (Huang et al., 2022). **(B)** Silver nanoparticles (nAg) are coated onto copper oxide microspheres (μCuO) through PDA; then, μCuO/nAg was loaded onto silk fibroin (SF) and spun onto the surface of SPEEK with polymerized PDA (SP-CuO/Ag) (Yan et al., 2020). **(C)** Schematic diagram of material synthesis and evaluation (Lyu, 2022). **(D)** Mechanism of Mg2^+^-PEEK scaffold osteogenesis and angiogenesis (Wei et al., 2023).

Currently, the effects of most PEEK angiogenic modification methods are significant, but they do not take into account the basic health status of the host. It is well known that the successful rate of bone defect implantation in diabetes mellitus is low, partly due to the relatively high possibility of infection in the diabetes microenvironment (Mangialardi et al., 2019). Another important reason is that patients with diabetes usually produce excessive ROS, resulting in oxidative stress in the microenvironment around the implant (Rendra et al., 2019). This phenomenon can have a negative impact on the release of VEGF from ECs, thereby affecting angiogenesis and nutrient supply around the implant, ultimately resulting in failed osseointegration and implant loosening. Therefore, loading antioxidants on the PEEK surface is an important means to promote angiogenesis in diabetes patients. Huang et al. (2022) first loaded TA, Pluronic F127 (PF127), and gentamicin sulfate (GS) on sulfonated PEEK (SP@ (TA-GS/PF)*3) by LBL. TA is a natural antioxidant with five diethylene glycol ester groups, which not only has high stability and activity but also has been proven to be an effective drug therapy for clearing ROS (Wu et al., 2021). The results obtained by Huang showed that H_2_O_2_ in the SP@ (TA-GS/PF)*3 group caused the weakest damage to human umbilical vein endothelial cells (HUVECs) and promoted the secretion of VEGF by HUVECs after injury. Immunohistochemical staining and analysis further revealed that SP@ (TA-GS/PF)*3 exhibited excellent angiogenesis-promoting ability by enhancing the expression of angiogenic-related cytokines (CD31 and vWF). In addition, osteoblast differentiation was also enhanced, which was beneficial to osseointegration under diabetic conditions. For patients with hypercholesterolemia or cardiovascular disease, stable and easily available small-molecule statins are widely used because of their function of protecting ECs. In recent years, it has been proven that statins can regulate angiogenesis and osteogenesis through miRNAs (Li et al., 2016). Sun et al. (2022) loaded simvastatin on SPEEK (concentrations 0.55, 1.1, and 2.2 mg/mL, groups SP-SimL, SP-SimM, and SP-SimH, respectively). The porous structure of sulfonated PEEK can achieve the sustained release of drugs, and this local drug delivery platform can suppress the systemic side effects of high-dose statins (such as hepatotoxicity and nephrotoxicity) and improve bioavailability (Liao, 2019). The drug release depended on the content and superficial area (Grassi et al., 2003), which was confirmed by the highest drug release rate of SP-SimM measured in the study. The drug concentration of SP-SimM was higher than that of SP-SimL, and the surface area was larger than SP-SimH. SP-SimM also had the strongest promoting effect on the formation of type-H vessels that regulated angiogenic–osteogenic coupling, which was contrary to the results of the miRNA–29cb^2^ knockout (miR29cb^2−/−^) mouse implantation experiment *in vivo*. This result provided direct evidence for the mechanism of simvastatin enhancing vascular regeneration. Previous studies by the author have shown that miRNA–29cb^2^ regulates type-H vessels to achieve angiogenesis and bone regeneration by targeting hypoxia-inducible factor-3α (HIF-3α), while other studies have suggested that HIF-3α and hypoxia-inducible factor-1β (HIF-1β) are in a competitively binding relationship. The hypoxic environment can stimulate the binding of hypoxia response elements (HREs) of HIF-1β and the promoter regions of angiogenic-related genes (VEGF and collagen-2α (Col-2α)) which can promote neovascularization (Duan, 2016; Zhang and Kong, 2023). In this study, SP and SP-SimM were implanted into WT and miR29cb^2−/−^ mice, and quantitative analysis of HIF-3α and HIF-1β around implants revealed that miR29cb^2^ knockout mice impaired the decreased HIF-3α expression in WT mice induced by simvastatin (Figure 9A). However, the mechanism of simvastatin on miRNA–29cb^2^ is very complex; thus, more analysis factors and observation time are needed to obtain more reliable evidence. The implant adjustment strategies for other common systemic diseases are worth in-depth research, which will provide great convenience for mixing with other systemic disease patients and broaden the indications for dental implants.

Drugs for systemic diseases usually have low stability, less amount of active drugs reaching the implant area after first-pass elimination, and damage to the liver and kidney. Therefore, topical modified implants can suppress their controversy in bone healing.

Bioactive metal elements (Cu and Mg) have multiple functions such as antibacterial activity, promoting angiogenesis, and enhancing bone regeneration. It is a novel modification method to assemble metal and catecholamine on the surface of PEEK. As a member of catecholamine, PDA can respond to pH by changing charge and degradation, so it can mediate the controlled release of metal elements (Yan et al., 2021). This on-demand release avoids high doses and premature elution of metal ions, thereby alleviating concerns about metal toxicity and bacterial drug resistance (Yan et al., 2020). The PDA structure contains abundant hydrophilic groups, such as amino, imino, and carboxyl groups, which can not only reduce the contact angle of PEEK but also serve as a bridge to bond the coating of metal ions (Wei et al., 2023). Most importantly, when the pH changes, amino groups in the PDA are protonated, which weakens its internal force, and the grafted metal elements are released and eluted (Yan et al., 2021). Therefore, PDA is an excellent adhesive candidate for binding metal elements. Cu has been proven to maintain the stability of the hypoxia-inducible factor-1α (HIF-1α) structure, which can simulate a hypoxia stimulate and then increase VEGF secretion and activate endothelial nitric oxide synthase (eNOS) to release NO which can accelerate the maturation of ECs (Murohara and Asahara, 2002; Urso and Maffia, 2015). Yan et al. (2020) coated silver nanoparticles (nAg) onto copper oxide microspheres (μCuO) through PDA; then, μCuO/nAg was spun onto the surface of SPEEK (Figures 9B). The study measured the production of NO, and the NO content of SP-CuO/Ag was more than twice that of the PEEK group, and the proliferation activity of HUVEC by the MTT method could also surpass that of the PEEK group on day 3. Furthermore, micro-computed tomography (micro-CT) showed that the coating was strong enough to withstand the stress generated by mechanical motion because there was no detachment after 12 weeks of implantation in the rabbit tibia. The author also invented another pH-responsive coating PDA-mediated co-deposition of citrate–copper nanoclusters (CCuNs) (Yan et al., 2021). It was unique in that the pH responsiveness was provided by PDA, and citrate also can induce angiogenesis, which could synergistically interact with Cu. The study found that the expression levels of HIF-1α, NO, VEGF, and iNOS in CCuNs were twice as high as those in Cu-loaded only samples, which might be related to the doubling of intracellular Cu levels caused by citrate (Finney et al., 2008). Due to angiogenesis and bone regeneration being coupled, there must be communication and crosstalk between ECs and osteoblastic cells (OBs) (Ramasamy et al., 2016). A co-culture system of adipose-derived mesenchymal stem cells (Ad-MSCs) and HUVECs simulating the real *in vivo* environment was established to evaluate the effect of CCuN-SPEEK on cell crosstalk. Compared with the single culture of Ad-MSCs, the secretion of ALP activity and collagen in the co-culture system increased by 20 times, while there was no calcium deposition on the surface of CCuN-PEEK. There is also a simple and efficient “one-pot” method for assembling Cu and DOPA on the surface of PEEK. Lyu (2022) directly coated a layer of PDA and CuII coordination complexes on the surface of PEEK (Figures 9C). If the concentration of metal ions exceeds a specific value, it will have cytotoxicity, and the critical concentration varies for different types of cells. The inductively coupled plasma–mass spectrometry (ICP-MS) results in the study showed that the highest concentration of Cu released by DA-CuII-coated PEEK was 0.2 ppm (not exceeding the toxicity level of most cells) (Kaplan and Maryon, 2016; Ning et al., 2016). In addition, all samples could promote the formation of tubes *in vitro* and the expression of angiogenic-related genes (VEGF, VEGF-A, and platelet-derived growth factor). There is increasing evidence that Mg^2+^, which is the fourth most abundant cation in the human body, can induce angiogenesis (Stegen et al., 2015). Wei et al. (2023) deposited Mg^2+^ on the surface of FDM-based PEEK using PDA as an adhesive. The pore size of PEEK scaffolds was 429 ± 37 μm, within the range of 300–500 μm, which was fully in favor of capillary ingrowth and substance exchange (Hutmacher, 2000). Type-H vessel is a special subtype of capillaries that couple angiogenesis and osteogenesis at both time and space levels in a bone homeostasis environment (Peng et al., 2020). High expression levels of CD31 and endomucin (CD31hiEMCNhi) are their characteristics (Xu et al., 2018). The study found that Mg^2+^-PDA PEEK significantly upregulated the expression of CD31 and endomucin (EMCN) compared to uncoated PEEK. More importantly, micro-CT of rabbit femoral condyles implanted with scaffolds showed that the diameter and volume fraction of blood vessels were the highest at all detection time points in the Mg^2+^-PDA PEEK group. The results of observing the number, thickness, and morphology of blood vessels in hard tissue sectioning under the microscope were consistent with these (Figure 9D demonstrates the mechanism of sample osteogenesis and angiogenesis). In recent years, PDA has been widely used for the functional modification of material surfaces due to its good biocompatibility and simple production process. Even though the material substrate has a complex shape with 3D pores, the dense coating of the PDA film will not be affected. The metal ions grafted on the surface of PDA promote angiogenesis at low concentrations and play a bactericidal role at high concentrations. Therefore, strictly controlling the critical concentration of metal ions is of utmost importance. The development of a coating on the surface of PEEK that releases appropriate concentrations will effectively avoid problems of bacterial resistance and metal toxicity.

Vascular regeneration is one of the prerequisite steps to promote the initial stability of osseointegration, and the cortical intraductal network within the cortical bone is composed of transversal Volkmann’s canals and longitudinal Haversian canals, which are intertwined with capillaries (Xie et al., 2014). Therefore, providing PEEK implants with the ability to generate blood vessels is crucial. Nevertheless, the chemical inertness of PEEK limits the reactive activity of ECs. At present, there are two main chains to enhance functional angiogenic response to PEEK implants: loading angiogenesis-related cytokines and stimulating endothelial cell autocrine VEGF. Obviously, the latter is a more advanced strategy because the loaded protein or polypeptide is easy to denature and inactivate after entering the complex internal environment and may also lead to ectopic vascularization. In addition, by combining modification methods to enhance the ability of angiogenesis and osteogenesis, solutions to the clinical application challenges of PEEK implants can be achieved, twice the result with half the effort.

### 4.7 Soft tissue adhesion

**FIGURE 10:**
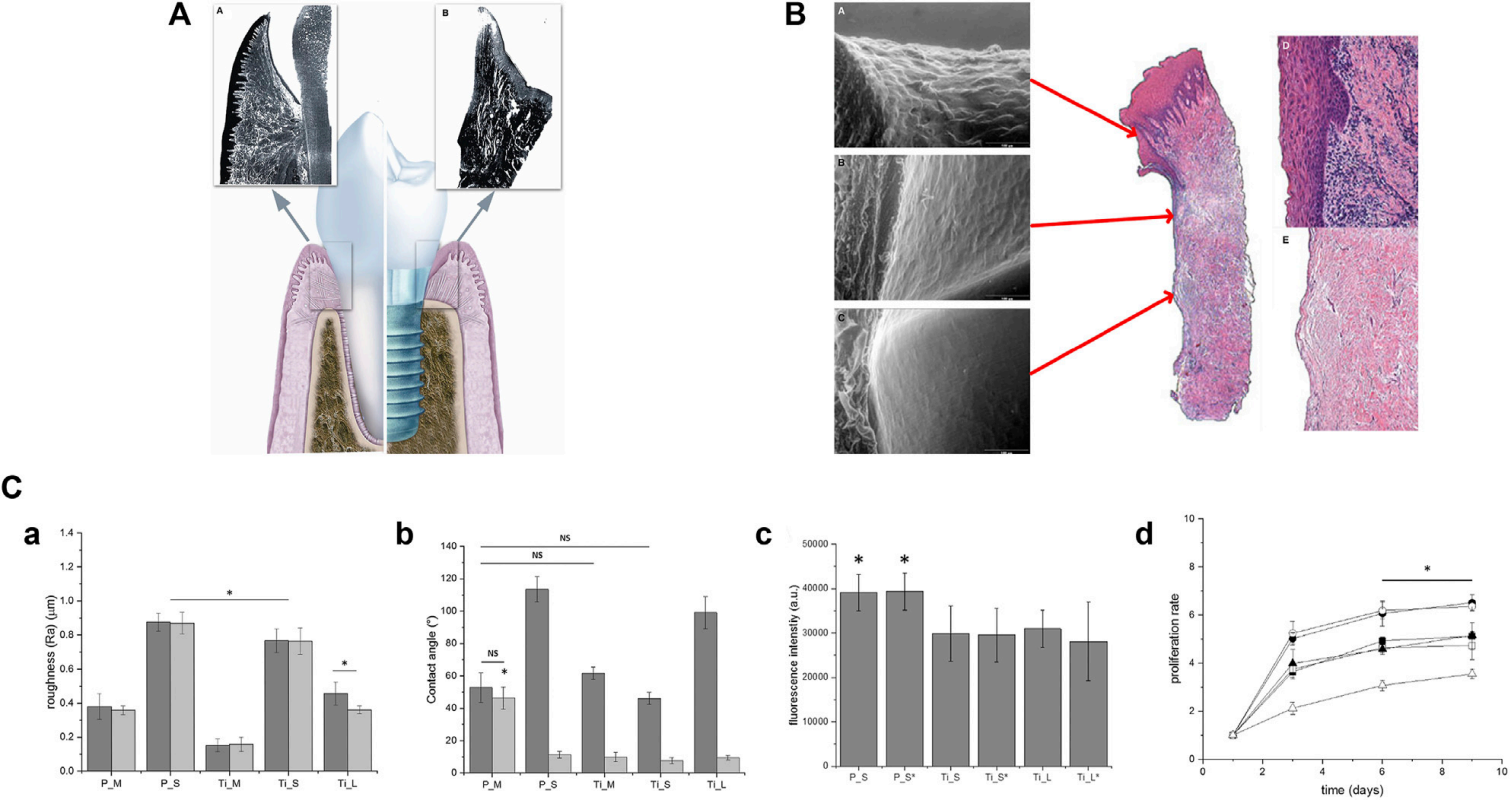
**(A)** Cross-section of the buccal and coronal part of the tooth and implant. Similar anatomical components (sulcular epithelium, junctional epithelium, and connective tissue) can be seen (Gheisarifar et al., 2021). **(B)** Histological sections of the peri-implant mucosa after 8 weeks of healing. (a) Sulcular epithelium. (b) Barrier epithelium. (c) Connective tissue (d). Epithelial layer. (e) Connective tissue (Ivanovski and Lee, 2018). **(C)** (a) Ra of Ti and PEEK samples before (gray) and after (light gray) air-plasma treatment. (b) Wettability of samples before (dark gray) and after (light gray) air-plasma treatment. (c) Cell adhesion of NIH-3T3 cells. (d) Proliferation of NIH-3T3 cells ((-P_S; -P_S; black dot-Ti_S; ◦-Ti_S; black triangle-Ti_L; triangle-Ti_L) (Porrelli et al., 2021).

The long-term stability of dental implants is achieved not only by combining with bone tissue but also by adhering to periodontal soft tissue to achieve biological sealing. The epithelial junction is the first barrier of tissue around the implant (the histological structure of the periodontal tissue around the implant is shown in Figures 10A, B), which can effectively prevent bacterial invasion and prevent the occurrence of implantitis. Peng et al. (2021b) compared the adhesion of human oral fibroblast (HOF) cells on the surface of CAD-CAM bare PEEK and traditional implant materials Ti6Al4V and Y-TZP. The pseudopodium structures of HOF cells were apparent on the surface of PEEK, which showed significant affinity. This is because the oxygen atoms with non-bonding pairs in the ether molecules of PEEK provide high polarity to increase the adhesion of cell receptors (integrins) on the cell membrane through adhesion proteins (fibronectin and collagen) (Ivanovski and Lee, 2018; Gheisarifar et al., 2021). However, due to the lack of biological activity of pure PEEK, which cannot be directly applied, scientists have explored many modification methods that can simultaneously improve the ability of osteogenesis and soft tissue adhesion.

It is known that laser etching, plasma treatment, and sandblasting can alter the surface morphology or introduce functional groups of PEEK and enhance its surface soft tissue cell adhesion. However, the three treatment methods have different effects. Femtosecond laser (FSL) uses a focused laser beam to form periodic features with micro/nano morphology on the material surface with high spatial and temporal resolution. For polymer materials, the maximum inhibition of surface oxidation is the greatest advantage of FSL. Xie et al. (2021) compared the effects of different powers of FSL (80 mW and 160 mW) on the behavior of soft tissue cells. SEM showed that unique submicro-nano structures were formed, and the number, adhesion, and proliferation of gingival epithelial (GE) cells were higher than those of pure PEEK; furthermore, the activity of 160FPK was more enhanced than that of 80FPK. At the same time, the adhesion, proliferation, and osteogenic-related gene expression of osteoblasts were also significantly enhanced. Consequently, laser etching plays an important role in increasing soft tissue sealing and bone regeneration. In order to compare the differences in the effects of laser etching and plasma treatment on human gingival fibroblasts (HGFs), Gheisarifar et al. (2021) treated PEEK with laser (PL), plasma (PP), and laser + plasma (PLP), respectively. It was found that laser etching had a stronger ability to improve HGF adhesion by increasing Ra, while plasma treatment had a better ability to increase HGF proliferation by reducing the water contract angle (WCA). However, some studies have shown that hydrophilicity is not conducive to the adhesion and diffusion of fibroblasts, which has been more clearly confirmed in the study of Porrelli et al. (2021). They proposed that, similar to laser etching, sandblasting also changed surface morphology to increase roughness. The adhesion of mouse embryonic fibroblast cells (NIH-3T3 cells) on the sandblasted PEEK surface was strongest and independent of hydrophilicity, while the proliferation of NIH-3T3 cells on the sandblasted Ti surface was strongest and only slightly dependent on hydrophilicity (Figure 10C). Therefore, roughness can affect the adhesion and proliferation of fibroblasts more than hydrophilicity. However, just as the study found that sandblasting PEEK could not inhibit biofilm formation, rough surfaces were also prone to bacterial adhesion, so multiple modification methods needed to be combined to achieve good bone integration of PEEK. Recently, a novel laser ablation (Synthegra^®^, Geass s. r. l., Italy) has been applied to Ti, which forms micro particles that can simultaneously promote eukaryotic cell adhesion and inhibit bacterial adhesion (Ionescu et al., 2018). The effect of this treatment on PEEK surface is still unknown. There are also some coating techniques that can promote soft tissue adhesion. Pang et al. (2021) deposited a 400-nm-thick nano-tantalum pentoxide (TP) coating on the surface of PEEK (PKTP) by vacuum evaporation (VE). The bioactive TP coating exhibited a 10-nm irregular protrusion on the surface of PEEK, which provided more sites for cell attachment. This was consistent with the enhanced adhesion and proliferation of HGEs on the PKTP surface. By the enhanced adhesion and proliferation of rBMSC, it could be seen that bone integration is also promoted. In addition to changing the morphology, preparing a tooth protein biomimetic coating can promote the adsorption of ECs. Periodontal tissue sealing is achieved by adhesion of epithelial cells to dentin and cementum. However, hemidesmosomes (HDs) and collagen I in dentin and cementum are in direct contact; furthermore, the basement membrane (BM) protein layer secreted by epithelial cells in HD is actually in direct contact with collagen I (Borradori and Sonnenberg, 1999; Bertassoni et al., 2012). Based on the aforementioned adhesion mechanism, Saad et al. (2022) prepared a layer of biomimetic collagen I coating on the surface of PEEK (Col-COOH-PEEK), with a Ti alloy as the control (Col-COOH-Ti). Through the results of label-free mass spectrometry (proteomics), it was found that the average protein score on Col-COOH-PEEK was five times that of Col-COOH-Ti, and the absorption of all BM proteins (laminin, nidogen, and fibronectin) was improved. Most importantly, laminins are proteins with the highest score and the only one with higher adsorption rates for Col-COOH-PEEK and Col-COOH-Ti than PEEK and Ti, which corresponded to the survival ability of keratinocyte epithelial cells on Col-COOH-PEEK being twice that of PEEK. That is, specific proteins have specific binding sites on collagen, and increasing the adsorption of specific proteins is of great significance for the adhesion of epithelial cells. The method of covalently coupling proteins may increase their long-term applicability. However, in order to ensure its stability, it is worth exploring the protein stability in an enzyme-containing environment. In addition, it will be of great significance to compare the binding strength with physically adsorbed proteins in the future.

All together, we provided a comprehensive introduction to the modification methods of biomechanical, anti-inflammatory, antibacterial, angiogenic, antioxidant, osteogenic and anti-osteoclastogenic, and soft tissue adhesion properties of PEEK, which can greatly expand the practical clinical application of PEEK. In order to apply it to the human body, we should also further understand the mechanism of implant osseointegration to help explore more strategies to comprehensively improve the biological activity of PEEK, which requires the joint efforts of medicine, chemistry, regenerative medicine, and other disciplines. Future research focuses are as follows: (1) *in vitro* testing of biocompatibility and chemical stability requires simulating the dynamic environment *in vivo*; (2) preclinical research should strictly select animal models and try to select large animal models for testing; (3) in order to simulate the natural oral environment, cyclic loading should be added to the stability assessment of bone integration. This can be achieved through three-dimensional finite element analysis mentioned in the following section; and (4) conducting randomized controlled clinical trials.

## 5 Discussion and conclusion

At present, a large number of experiments have confirmed that PEEK can overcome the limitations brought about by biological inertness through surface or blending modification. This makes PEEK frequently seen in the biomedical field, such as spinal interbody fusion cages, artificial hip joints, and artificial knee joints in spinal surgery and orthopedics, as well as dental removable partial dentures, fixed dentures, implant abutments, and orthodontic arch wires in stomatology. However, it cannot be ignored that PEEK dental implants discussed in this article seem to be limited to the initial exploration stage and lack effective clinical implantation research and data. In other words, so far, no research has applied modified PEEK to humans, and only a small portion has studied its osseointegration in dog jaws. A considerable number of studies have reported successful cases of PEEK as other jawbone implant materials. EL Morsy et al. (2020) used PEEK as a barrier material for guided bone regeneration, while Li et al. (2022b) used PEEK for the treatment of mandibular segmental bone defects. Due to the excellent biocompatibility, mechanical properties, and processability of PEEK, there were no postoperative complications such as infection or displacement due to bearing chewing power in both studies. In the past decade, only two studies reported the effectiveness of PEEK dental implants. Marya et al. (2011) demonstrated three cases of PEEK dental implants. The author believed that the implants had the potential for osseointegration during the 6-month follow-up period. However, the report had a small number of participants and did not introduce evaluation methods. On the contrary, the report by Khonsari et al. (2014) showed three cases with severe postoperative infections, resulting in failed osseointegration and, ultimately, implant loosening. In summary, it still takes some time to translate the results created in the laboratory into practical clinical treatment, and this process may face many challenges, for example, the stability of surface chemical coating and the activity maintenance *in vivo*, whether degradable components can be accurately released at the target site, whether implants can play a role in the constantly changing oral environment, and the differences in habits and bones between patients. These issues are both crucial and difficult to solve. Therefore, animal models with strict standards should be established for testing to maximize the homogeneity of preclinical analysis.

Within the scope of this review, it can be concluded that PEEK is expected to replace traditional dental implant materials. Its aesthetics and low stress shielding make it have greater application advantages in special patient groups. In addition, the stress distribution in the bone tissue around PEEK is not inferior to traditional implants and is even more suitable for patients with poor bone conditions. Furthermore, we summarized a series of parameters to optimize the performance of 3D-printed PEEK with complex porous structures. Finally, different functionalization strategies are proposed to enable patients to benefit from non-metallic implants. Currently, PEEK research studies usually lack large-scale animal testing and randomized controlled clinical trials. Combining with the complex specific dynamic environment of the human body, future research should focus on animal experiments and clinical research with cyclic loading and long observation time and combine multidisciplinary efforts to achieve a broader application of PEEK.
